# Effects of electro-mechanical uncouplers, hormonal stimulation and pacing rate on the stability and function of cultured rabbit myocardial slices

**DOI:** 10.3389/fbioe.2024.1363538

**Published:** 2024-04-05

**Authors:** V. Baron, S. T. Sommer, D. J. Fiegle, A.-K. M. Pfeuffer, R. Peyronnet, T. Volk, T. Seidel

**Affiliations:** ^1^ Institute of Cellular and Molecular Physiology, Friedrich-Alexander-Universität Erlangen-Nürnberg, Erlangen, Germany; ^2^ Institute for Experimental Cardiovascular Medicine, University Heart Center Freiburg, Bad Krozingen, Germany; ^3^ Faculty of Medicine, University of Freiburg, Freiburg, Germany

**Keywords:** contractility, biomimetic chambers, organotypic culture, multicellular preparations, reperfusion injury

## Abstract

**Introduction:** Recent advances have enabled organotypic culture of beating human myocardial slices that are stable for weeks. However, human myocardial samples are rare, exhibit high variability and frequently originate from diseased hearts. Thus, there is a need to adapt long-term slice culture for animal myocardium. When applied to animal cardiac slices, studies in healthy or genetically modified myocardium will be possible. We present the culture of slices from rabbit hearts, which resemble the human heart in microstructure, electrophysiology and excitation-contraction coupling.

**Methods:** Left ventricular myocardium from New Zealand White rabbits was cut using a vibratome and cultured in biomimetic chambers for up to 7 days (d). Electro-mechanical uncoupling agents 2,3-butanedione monoxime (BDM) and cytochalasin D (CytoD) were added during initiation of culture and effects on myocyte survival were quantified. We investigated pacing rates (0.5 Hz, 1 Hz, and 2 Hz) and hormonal supplements (cortisol, T3, catecholamines) at physiological plasma concentrations. T3 was buffered using BSA. Contractile force was recorded continuously. Glucose consumption and lactate production were measured. Whole-slice Ca^2+^ transients and action potentials were recorded. Effects of culture on microstructure were investigated with confocal microscopy and image analysis.

**Results:** Protocols for human myocardial culture resulted in sustained contracture and myocyte death in rabbit slices within 24 h, which could be prevented by transient application of a combination of BDM and CytoD. Cortisol stabilized contraction amplitude and kinetics in culture. T3 and catecholaminergic stimulation did not further improve stability. T3 and higher pacing rates increased metabolic rate and lactate production. T3 stabilized the response to β-adrenergic stimulation over 7 d. Pacing rates above 1 Hz resulted in progredient decline in contraction force. Image analysis revealed no changes in volume fractions of cardiomyocytes or measures of fibrosis over 7 d. Ca^2+^ transient amplitudes and responsiveness to isoprenaline were comparable after 1 d and 7 d, while Ca^2+^ transient duration was prolonged after 7 d in culture.

**Conclusions:** A workflow for rabbit myocardial culture has been established, preserving function for up to 7 d. This research underscores the importance of glucocorticoid signaling in maintaining tissue function and extending culture duration. Furthermore, BDM and CytoD appear to protect from tissue damage during the initiation phase of tissue culture.

## 1 Introduction

Organotypic culture of myocardial slices from humans and animal models has been advanced during the last few years and is getting attention for its potential in basic and translational cardiac research (Fischer et al., 2019; Ou et al., 2019; Qiao et al., 2019; Watson et al., 2019; Perbellini and Thum, 2020). Although the short-term study of slices dates back to the 1960s (Mokler and Mathur, 1968), structure and function in human ventricular (Fischer et al., 2019) and atrial (Klumm et al., 2022; Amesz et al., 2023) tissues can now be preserved for up to several weeks, allowing the investigation of long-term effects of experimental procedures without the dedifferentiation or functional decay commonly present in primary cardiomyocyte culture (Banyasz et al., 2008). Compared to cell culture, native tissue culture has several key advantages: avoidance of potential side effects associated with enzymatic and/or cell isolation, and a native multi-cellular co-culture, akin to that found *in vivo*. Cardiomyocytes remain in their native environment, where cell-cell and cell-matrix interactions are largely preserved. Furthermore, direct and indirect interactions with and between non-myocytes, for example, fibroblasts, are preserved and can be investigated (de Boer et al., 2009). At the same time, it is possible to analyze the metabolites, cytokines and other substances produced by the tissue because they accumulate in the culture medium. Furthermore, long-term myocardial slice culture can be used in conjunction with stem-cells or stem-cell derived cardiomyocytes and gene editing (Moretti et al., 2020; Poch et al., 2022). In summary, these features allow for many already demonstrated and conceivable applications and may provide new important insights into basic cardiac physiology and pathology.

As it can be difficult to obtain human tissue for research due to its limited availability, and even more so for healthy tissue from human donor hearts, animal models offer a valuable alternative. A recent study used cardiac slices from pigs to demonstrate the feasibility of cardiotoxicity screening (Shi et al., 2023). However, while pig hearts are well comparable to human hearts, there are functional differences, such as a low I_to_ current, and high maintenance and animal costs (Milani-Nejad and Janssen, 2014). Small rodent hearts, on the other hand, differ significantly from human hearts in several aspects (Milani-Nejad and Janssen, 2014), for example, in action potential duration or contraction kinetics, and, importantly, only few slices can be obtained for slice culture from one animal. In contrast, cardiac models from animals of intermediate size, such as guinea pig or rabbit, appear to have more favorable properties. For instance, the action potential duration and shape as well as Ca^2+^ cycling and excitation-contraction coupling are much closer to humans in guinea pigs and rabbits than in rats or mice. Generally, hearts are larger and heart rate is lower in rabbits than in guinea pigs. Moreover, rabbit cardiomyocytes express I_to_, which can hardly be detected in guinea pigs (Joukar, 2021). Thus, while both rabbit and guinea pig models have been used extensively in cardiac research (Janse et al., 1998; Schram et al., 2002), rabbit hearts provide more material for cardiac slices and resemble human hearts more closely in several functional aspects (Nerbonne, 2000; Hornyik et al., 2022). Furthermore, rabbit models offer advantages for the research of various heart diseases (Pogwizd and Bers, 2008; Hornyik et al., 2022). Therefore, they could represent a useful, cost-effective alternative to pig hearts without compromising the relevance and transferability of the results to human hearts. However, although rabbit myocardial slice culture has been used in research (Watson et al., 2019), we are not aware of systematic studies to extend the useful life span in culture.

In this study, we describe optimization of the culture of rabbit ventricular slices. This was required because protocols designed for human tissue slices gave rise to contracture at the beginning of culture experiments. Additionally, slices often decayed after few days or displayed unstable contraction amplitude. These problems are worsened by the lack of consensus in the field about ideal culture conditions, especially with respect to medium supplements and pacing rate during culture. We present enhanced culture conditions to mitigate initial damage and evaluate the effects of pacing frequency and commonly used hormonal additives on culture stability and viability. Using these protocols, viability of rabbit ventricular slices could be maintained for 1 week. Finally, we demonstrate techniques for advanced functional assessments, such as action potential or Ca^2+^ transient recordings in intact cardiac slices to assess the stability of essential myocardial functions and show that Ca^2+^ signals are nearly unaltered after 7d in culture.

## 2 Materials and methods

### 2.1 Tissue acquisition

All experiments were approved by the local animal care and use committee. Female New Zealand White rabbits (age 10–16 weeks, weight 2.0–3.5 kg) were premedicated with ketamine (25 mg/kg) and xylazine (5 mg/kg) *via* intramuscular injection. After 10 min, anesthesia and analgesia were verified by the absence of pain reactions and pentobarbital sodium injected into the dorsal ear vein (200 mg/kg). This caused deep anaesthesia and death from cardiac arrest. Swiftly afterwards, thoracotomy and excision of the heart followed. The heart was transferred to cold storage solution and then transported to the lab within 15–30 min. Storage solution contained in mmol/L: 80 potassium glutamate, 10 NaCl, 30 butanedione monoxime, 50 sucrose, 25 KH_2_PO_4_, 5 MgSO4, 1 CaCl_2_, 1 allopurinol, 5 adenosine, 5 glutathione, pH = 7.4.

### 2.2 Preparation of ventricular tissue slices

After arrival of the rabbit heart in the laboratory it was transferred into a culture dish (⌀10 cm) filled with cold (4 °C) storage solution (the same solution as used for transport and storage). All preparation steps were carried out on a laminar flow workbench on a cooled surface at 4°C–8°C. The free wall of the left ventricle was prepared and divided into four quadrants (surface approx. 8 mm × 8 mm). Two tissue blocks at a time were embedded in low-melting point agarose and mounted with the epicardium facing down on the vibratome as described in detail previously for human tissue (Fischer et al., 2019; Hamers et al., 2022). Myocardial slices of 300 µm thickness were cut with the vibratome (Leica VT1200, Germany) with a forward movement speed of 0.05 mm/s, 80 Hz blade oscillation frequency and 1.5 mm horizontal razor blade amplitude in parallel to the epicardial plane. As soon as the first two tissue blocks were processed, the remaining blocks were embedded in agarose and sliced accordingly. Subendocardial tissue slices lacking a continuous myocyte alignment (typically the first three to four slices) were discarded. Slices with a uniform cardiomyocyte orientation were trimmed with a scalpel to approx. 6 mm × 6 mm length and width. Cardiomyocyte orientation was determined macroscopically or, if not readily visible, by transmitted light microscopy. Trapezoid polyether holders (InVitroSys, Germany) were then attached by surgical tissue adhesive (Surgibond, SMI, Belgium) to two opposing sides of the slice, such that they were connected by the longitudinal myocyte axes (Fischer et al., 2019; Hamers et al., 2022). The slices were stored in cold (4°C) storage solution for max. 60 min until they were mounted in the culture chambers. One rabbit heart yielded up to 30 tissue slices.

### 2.3 Culture of ventricular tissue slices

Using MyoDish culture systems (MD-1.0, InVitroSys, Germany), the tissue slices were mounted into chambers with culture medium (M199, Sigma, M4530), supplemented with insulin (10 ng/mL), transferrin (5.5 μg/mL), selenium (6.7 ng/μL), β-mercaptoethanol (50 µM), penicillin (100 units/mL)/streptomycin (0.1 mg/mL), as used for human myocardial long-term culture (Fischer et al., 2019). Electromechanical uncoupling agents were added in different combinations: 30 mM 2,3-butanedione monoxime (BDM), 5 µM cytochalasin D (CytoD), 10 µM blebbistatin, 50 μM N-benzyl-p-toluene sulphonamide (BTS). The culture system including the chambers was kept in a laboratory incubator providing 5% CO_2_, 37°C temperature and 80% humidity. A diastolic load of 1,500 µN was applied to each slice immediately after starting culture. If the initial medium contained uncouplers, the medium was removed after 15–20 min, the slice washed in 800 µL agent-free medium for a few seconds, and then 2.4 mL final culture medium were added. During the first 48 h of culture, all slices were paced at 0.5 Hz. The applied current was 50 mA, pulse width 3 ms, followed by a 1 ms pause and a 3 ms pulse of opposite polarity. The applied voltage necessary to reach the specified current ranged from 7–9 V. If not otherwise stated, medium was exchanged every 48 h. Assuming an evaporation of 100 µL per 24 h, 1.6 mL of the medium in the chamber were removed and 1.8 mL fresh medium added. The slices were assigned to experimental groups after 48 h in culture, i.e., during the first medium exchange. Slices were cultured for up to 7 days.

### 2.4 Medium additives

Different hormonal additives were added alone or in combination to the culture medium described above to obtain free concentrations of 20 nM cortisol and 10 pM T3, which are close to normal free plasma levels in rabbits (Raekallio et al., 2002; Mustafa et al., 2008; Habeeb et al., 2018) and humans (Marwaha et al., 2013; Dichtel et al., 2019). Another goal was to test the effects of baseline adrenoceptor stimulation. However, we refrained from using adrenaline, noradrenaline or isoprenaline due to their short plasma half time *in vivo* and presumed instability in culture medium due to spontaneous and enzymatic degradation (Jones and Robinson, 1975; Hjemdahl, 1993; Palombo et al., 2022). We therefore used drugs with longer half-times: denopamine (β_1_ receptor) (Kino et al., 1983), salbutamol (β_2_ receptor) (Goldstein et al., 2003) and phenylephrine (α_1_ receptor) (Hengstmann and Goronzy, 1982). Concentrations were chosen to result in the stimulation of the respective receptors one would assume from resting plasma levels of 1–2 nM adrenaline and 5–10 nM noradrenaline (Hamelberg et al., 1960; Abd-Allah et al., 2004). Denopamine has approx. 1/2 the potency of adrenaline and noradrenaline (Naito et al., 1985), phenylephrine approx. 1/10 the potency of noradrenaline (Mohta et al., 2019) and salbutamol a similar potency as adrenaline (Anakwe and Moger, 1984). Considering albumin binding (Nagao and Nakajima, 1989; Rich et al., 2001; Volpp and Holzgrabe, 2019) and degradation of these substances over 24–48 h, we chose concentrations of 50 nM for each.

To achieve stable concentrations of free T3, 0.5% BSA was added as a buffer with a published affinity constant of T3 to albumin of 6.2×10^5^ M^−1^ (Yabu et al., 1987), translating into a dissociation constant K_D_ = 1.62 µM. From this, we calculated the concentration of total T3 for a given concentration of albumin and free T3 as follows:

$${\left[T3\right]}_{\text{total}}={\left[T3\right]}_{\text{free}}+{\left[\text{albumin}\right]}_{\text{total}}\;•\;{\left[T3\right]}_{\text{free}}/\left(K_{D}+{\left[T3\right]}_{\text{free}}\right)$$

The calculation yields a necessary total T3 concentration of ≈460 pM to obtain a free T3 concentration of 10 pM in the presence of 0.5% BSA. Because cortisol is also bound to BSA (Mueller and Potter, 1981), the total concentration of cortisol was increased to 35 nM in media containing 0.5% BSA. We verified the free concentrations of free T3 and cortisol in culture medium 24 h after medium exchange with LIAISON^®^ FT3 (DiaSorin, 311,531) and LIAISON^®^ Cortisol (DiaSorin, 311,861) test kits.

### 2.5 Contraction analysis

Contraction analyses were performed 24 h after mounting into chambers and then every 24 h until the end of the experiment as described previously (Abu-Khousa et al., 2020; Klumm et al., 2022). The duration from the onset of contraction, defined as the point where the contraction force reaches 10% of its peak value, to the peak itself was defined as time to peak force (TTP). The duration required to decrease from the peak force to 10% of the peak value was defined as time to relaxation (TTR).

### 2.6 Ca^2+^ imaging and signal processing

Following a protocol described previously (Klumm et al., 2022), Ca^2+^ imaging was performed by placing a slice into a culture chamber with a glass bottom and loading the slice with Calbryte 520-AM Ca^2+^ indicator (AAT Bioquest, Sunnyvale, CA) at 10 μmol/L and 0.1% Pluronic Acid F127 (Biotium, United States) and incubated for 20 min at 37°C in a pH-buffered solution containing in mM: 138 NaCl, 4 KCl, 2 CaCl_2_, 1 MgCl_2_, 0.33 NaH_2_PO_4_, 10 HEPES, 10 glucose, pH = 7.4. During imaging, slices were superfused with the same solution at 37°C at 250 mL/h. The excitation wavelength spectrum was 460–500 nm. The emitted light was filtered with a band pass 510–585 nm and detected with a photomultiplier (IonOptix) at a sampling rate of 1 kHz. The recorded contraction and Ca^2+^ signals were synchronized using the electrical stimulation signal. Electrical stimulation was applied via the same graphite electrodes used during culture (field stimulation).

For display, the raw Ca^2+^ signal was filtered with a moving median filter (window size 3), followed by a moving mean filter (window size 3) and a moving geometric mean filter (window size 3). For calculation of Ca^2+^ transient (CAT) parameters (amplitude and 90% duration, CATD90), the raw signal was filtered with a moving median filter (window size 9), followed by a moving mean filter (window size 9). After filtering, local peaks were detected and the minimum signal (S_min_) in the interval between each peak and its preceding peak was defined as baseline signal. The value of the signal at the peak position was defined as the maximum (S_max_). The amplitude (A) of each CAT was calculated by subtracting the maximum from the respective minimum (A = S_max_ − S_min_). CATD90 was calculated as the duration from the first time point after the electrical stimulation where signal intensity was ≥0.1 A to the first time point after the peak where signal intensity was ≤0.1 A.

### 2.7 Action potential recordings

Transmembrane potentials were recorded using sharp microelectrodes filled with 3 M KCl and with a tip resistance between 4 and 15 MΩ KCl. Borosilicate capillaries with filament (GC100F-15, Harvard Apparatus, Holliston, MA, United States) were pulled using a P-97 puller (Sutter, Novato, CA, United States). Electrodes were connected to a bridge amplifier BA-01X (NPI Electronic, Tamm, Germany) and positioned using a SensApex micromanipulator (Sensapex, Oulu, Finland). Recorded potentials were digitized at 50 kHz and low-pass filtered at 10 kHz using a Labview interface (National Instruments, Austin, TX, United States). Slices were paced by a stainless steel concentric bipolar electrode (core electrode diameter 100 μm, Science products, Hofheim am Taunus, Germany) driven by a MyoPacer stimulator (IonOptix, Westwood, MA, United States). Bipolar stimulation pulses were applied with a duration of 1–4 ms and a voltage that was twice the threshold. A point stimulator was used to be able to distinguish clearly the stimulation artefact from the beginning of the action potential. Membrane potentials recorded in the culture chambers during constant superfusion with a modified Tyrode’s solution containing (in mM): 140 NaCl, 5.4 KCl, 1 MgCl_2_, 10 HEPES, 10 glucose, 1.8 CaCl_2_, pH 7.4 at 37°C and bubbled with O_2_.

### 2.8 β-adrenergic response

The β-adrenergic response was assessed using 100 nM isoprenaline on days 2 and 6 of the culture. As the opening of the incubator disturbed the CO_2_ and temperature equilibrium, we waited a minimum of 5 min and until incubator CO_2_ level reached 5% again. Contraction amplitude was measured after equilibration and when a steady state level had been reached, which was the case after the 5-min waiting time. Afterwards the medium was completely exchanged with fresh medium.

### 2.9 Viability assays

#### 2.9.1 MTT assay

Cellular viability in tissue slices were measured using a (3-(4,5-dimethylthiazol-2-yl)-2,5-diphenyltetrazolium bromide (MTT, Sigma, M2128) assay (Brandenburger et al., 2012). We used 15 slices with differing contraction amplitudes collected after different culture durations. The slices were washed twice with 1 mL MTT buffer (in mM: 130 NaCl, 5.4 KCl, 1.8 CaCl_2_, 1 MgCl_2_, 0.33 NaH_2_PO_4_, 10 glucose, 23 NaHCO_3_, 30 BDM). Afterwards 2 mL MTT buffer containing 0.5 mg/mL MTT were added into the culture chamber. The slices were stained in an incubator (37°C, 5% CO_2_ and >80% relative humidity) with permanent agitation for 20 min on the MyoDish system. Afterwards the stained slices were washed once with 2 mL MTT buffer. The stained slices were frozen in liquid nitrogen and stored at −80°C. Quantification was achieved by absorbance measurement of MTT stain in DMSO and normalized to protein mass which was assessed using Bradford assays (Bio-Rad) (Klumm et al., 2022).

#### 2.9.2 Dextran uptake assay

A Dextran uptake assay in combination with ryanodine receptor (RyR) immunofluorescence was performed to assess viability of the tissue slices according to a published method (Pfeuffer et al., 2023). Briefly, dextran (3 kDa) conjugated to FITC (Thermo Fisher, D3306) at a concentration of 2 mg/mL was incubated for 10 min at 37°C and 5% CO_2_ under continuous rocking on the MyoDish culture system platform. The slices were immediately fixed with 2% paraformaldehyde (PFA) in phosphate-buffered saline (PBS) for 10 min and then washed three times with PBS for 5 min each. Subsequently, slices were incubated with primary antibody against cardiac RyR (IgG1, mouse, C3-33, Thermo Fisher, Braunschweig, Germany) 1:200 in blocking solution (BS: 5% NGS, 5% BSA, 0.25% Triton-X in PBS) for 4 h at RT or overnight at 4°C, washed three times with PBS for 5 min and incubated with the secondary antibody goat anti-mouse IgG1 AF-555 (Thermo Fisher A-21127) 1:400 in BS for 3 h at RT. After washing, the slices were incubated with wheat germ agglutinin (WGA-AF-647 Thermo Fisher W32466) 40 μg/mL and DAPI (3665, Roth, Karlsruhe, Germany) 1.67 μg/mL in PBS for 3 h. The slices were mounted with Fluoromount G (00-4958–02, Thermo Fisher; F4680 Sigma-Aldrich, Darmstadt, Germany) on a glass microscope slide, covered with a coverslip and equilibrated for 3–7 days at 40%–45% humidity.

### 2.10 Immunofluorescent staining and confocal microscopy

From dextran-/RyR-stained slices we acquired two-dimensional confocal tile scans of 12 × 12 tiles with a pixel size of 0.1 × 0.1 µm^2^ and 10% tile overlap with a Zeiss LSM780, using a 63× oil immersion lens. Stitching of the tiles was performed with the microscope software package (ZEN). All slices were imaged at the same depth of approximately 25–30 µm below the coverslip. A two-track imaging protocol was used for slices with 4 channels, with parallel excitation of DAPI and AF-555, using laser wavelengths of 405 nm and 561 nm, respectively, and a second track with laser wavelengths of 488 nm and 633 nm to excite FITC and AF-647.

For microstructural analyses of myocytes and the quantification of the extracellular matrix, freshly cut and cultured slices were fixed in 2 mL 2% PFA in PBS was added for 10 min. Afterwards the slices were washed 3 times for 3 min with PBS. WGA conjugated to AF647 and DAPI were used to stain the glycocalyx and extracellular matrix proteins, including the transverse-axial tubular system (TATS), and the nuclei, respectively, as described (Abu-Khousa et al., 2020; Shi et al., 2023). Afterwards, the slices were mounted onto a microscope slide with Fluoromount G. After 3–7 d of drying the slices were imaged with Zeiss LSM780 with laser wave lengths of 405 nm and 633 nm. Three 3D stacks of dimensions 100 × 100 × 25 μm^3^ and one 2D tile scan (≈1 × 1 mm^2^) were acquired from each slice.

### 2.11 Image processing and analysis

The 3D confocal volumes were processed based on a modified pipeline developed previously (Greiner et al., 2022). Initially, WGA signals were examined and cropped to ensure complete XY planes of cardiomyocytes at the beginning of the image volume and to exclude areas at the end of the stack where image quality was compromised by scattering. After cropping, the average depth of each volume was 19.6 μm. Instead of the custom 3D Unet in the original pipeline, we used the nnU-Net framework (Isensee et al., 2021) to predict cardiomyocyte boundary probabilities given the cropped WGA volume. The predicted boundary probabilities were thresholded at 0.5, and a distance-based watershed was calculated on the resulting binary mask. A multicut formulation (Pape et al., 2017) was used to generate an initial agglomeration of the watershed’s supervoxels. The final semi-automatic agglomeration was done using a custom-developed graphical user interface, allowing for merging, unmerging, new supervoxel generation, and manual painting. All segmentations were proofread by a researcher. The initial neural network was trained on the data of (Greiner et al., 2022), then continually retrained on newly proofread segmentations.

Cardiomyocyte width and depth were calculated using the second and third largest dimensions of the oriented bounding box of each segmented cardiomyocyte. The determination of cardiomyocyte length was omitted, as they were frequently cut at the volume boundaries, rendering this measure influenced by the imaging plane relative to cardiomyocyte orientation. Furthermore, we calculated volume fractions based of cardiomyocytes and the inter-cardiomyocyte space. The inter-myocyte space was further refined into a WGA-positive and WGA-negative fraction using a histogram-based threshold of the WGA signal (Seidel et al., 2016) (mode +1 standard deviation). The WGA-positive intermyocyte volume correlates with the amount of extracellular matrix and, thus, with fibrotic remodeling as it labels fibrotic tissue constituents, while the WGA-negative intermyocyte volume correlates with extracellular clefts and interstitial fluids. Volume measures were averaged over all volumes originating from the same slice.

The TATS was segmented using the same histogram-based threshold of the WGA signal (mode +1 standard deviation) and the cardiomyocyte reconstruction. The distance of the cardiomyocytes’ cytoplasm to the TATS was calculated by the 3D exact Euclidean distance (van der Walt et al., 2014). For the calculation of average TATS distances and cardiomyocyte dimensions, cell fragments smaller than 3,000 μm^2^ were excluded. All cell-based measures are calculated per cardiomyocyte, averaged within each volume, and then further averaged across all samples from the same tissue slice.

Image processing and analysis of the dextran uptake assay was performed according to a recently published method (Pfeuffer et al., 2023).

### 2.12 Glucose and lactate measurements, metabolic rate estimation

Glucose and lactate concentrations in the medium were determined using a blood gas analysis device (epoc^®^, Siemens) at three timepoints: immediately after medium exchange at day 4, and then 24 h and 48 h after the initial measurements. For the analysis 200 µL culture medium was drawn from culture chambers and either directly analyzed or stored for up to 1 week at −80°C before analysis. From the obtained concentrations of glucose and lactate in the medium, we calculated the amounts of metabolized glucose and produced lactate during 24 h and 48 h, taking into account the volume of medium in the culture chambers after the medium exchange (2.6 mL), the volume removed for analysis (200 µL for each measurement) and the evaporation. Evaporation was estimated from the increase in Na^+^ and Cl^−^ concentrations. Metabolic rate in mW/g and percentage of aerobic metabolism were calculated from the molar ratio of lactate produced and glucose metabolized, assuming slice dimensions of 5 × 5 × 0.25 mm^3^. These dimensions were assumed slighty smaller than the nominal size of the slices, assuming that myocytes at the borders were damaged due to cutting and trimming. We assumed 6 mol ATP per mol glucose during anaerobic glycolysis, producing 2 mol lactate, and additional 25 mol ATP per mol glucose if subjected to oxidative phosphorylation, indicated by the removal of 2 mol lactate. We presumed an energy turnover of 57 kJ per mol ATP.

### 2.13 Statistics

If not otherwise indicated, data are presented as mean ± standard error. Where applicable (multiple measurements within the same slice), a paired t-test was used. Otherwise, an unpaired two-tailed t-test was applied. Assumptions about (un)equal variance are stated in the respective figure legends. If equal variances were assumed, this assumption was checked with Levene’s test for equal variances. When showing multiple comparisons within one chart or figure, the multiple comparison correction of *p* values according to Holm-Bonferroni was applied. The level of significance was set to α = 0.05.

We employed linear regression to model MTT absorbance as a function of contraction amplitude (Figure 3) using the ‘fitlm’ function within MATLAB (Mathworks, version 2023a). The model was assessed using an F-test vs. a degenerate model with only a constant term. Further, we used linear regression to model glucose and lactate concentrations as a function of time (Figure 8) with interaction terms for T3 supplementation and pacing frequencies (concentration ∼1 + time + time:pacing frequency + time:T3). In this model, ‘time’ and ‘pacing frequency’ are continuous variables, ‘T3’ indicates the presence of supplementation as a binary variable. We used the function ‘OLS’ from the Python package ‘statsmodels’ (Seabold and Statsmodels, 2010), version 0.13.0. The model was assessed using an F-test vs. a degenerate model with only a constant term. The significance of parameters was assessed using a two-tailed t-test with the assumption of equal variance.

## 3 Results

### 3.1 Rabbit ventricular slice culture

**FIGURE 1:**
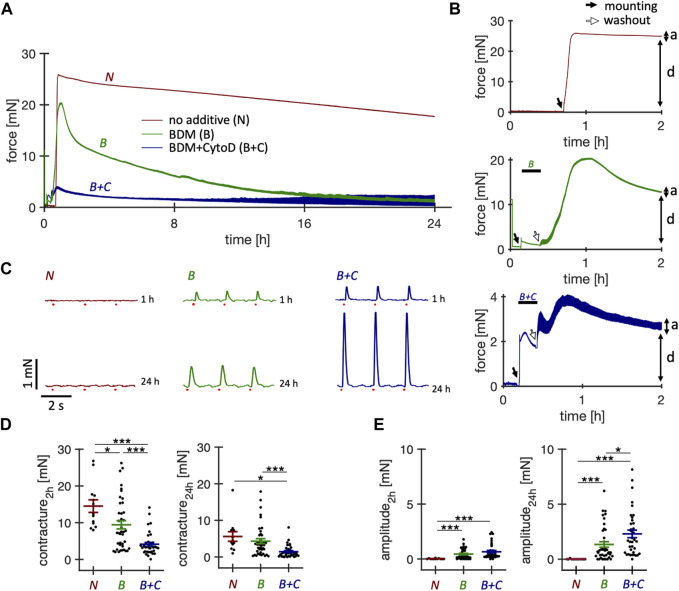
Effect of short-term treatment with electromechanical uncouplers on the contractile function of cultured rabbit ventricular slices. Behavior of rabbit ventricular slices immediately after initiation of culture, either without additives or with supplementation for 15 min of butanedione monoxime (BDM) or BDM + Cytochalasin D (CytoD). All slices were constantly paced at 0.5 Hz. **(A)** Example force recordings of the first 24 h obtained from slices of the same heart, brought into culture without supplement (*N*, *red*), with 30 mM BDM (*B*, *green*), or with 30 mM BDM +5 µM CytoD (*B + C*, *blue*). After 15–20 min, BDM and BDM + CytoD were washed out. **(B)** Magnified views of the first 2 hours. A preload of 1.5 mN was applied immediately after placing the slices into the cultivation chambers, indicated by solid arrows. Note that culture was started at slightly different times. The force traces before the solid arrows resulted from agitation of the culture medium. Washout of BDM or BDM + CytoD is indicated by open arrows. Also note the different scaling of the y-axis. Passive diastolic force **(D)** is indicated as well as active systolic force **(A)**. **(C)** Force recordings (6 s displayed) after 1 h and after 24 h in culture of contractions elicited by field stimulation (red dots). Here, we optimized the culture conditions of rabbit ventricular slices with the aim of improving initial survival immediately after placement in culture and long-term stability, using physiological pharmacological or hormonal stimulations that do not interfere with experiments performed in addition to these supplements or cause non-physiological activation of specific signaling pathways. **(D)** Diastolic contracture, measured as maximum diastolic force in addition to the applied preload of 2 mN during the first 2 h after and at 24 h after the onset of tissue culture. **(E)** Contraction amplitudes at 2 h and 24 h after onset of tissue culture. Statistics: n = 13/4 (*N*), 40/6 (B), 36/4 (*B + C*) slices/hearts, respectively. *p < 0.05, ***p < 0.001, unequal variance, two-tailed t-test.

In our initial experiments, we used protocols published for human slices (Fischer et al., 2019; Abu-Khousa et al., 2020; Hamers et al., 2022) and mounted rabbit myocardial slices in culture chambers containing M199-based culture medium prewarmed to 37°C. However, this repeatedly resulted in immediate and sustained contracture of the slices and no detectable response to electrical stimulation. This led us to introduce electromechanical uncoupling agents for protection during the first 15–20 min in culture (Figure 1). We examined a total of 103 tissue slices and monitored their force development for the first 24 h. We investigated the uncoupler BDM (n = 40 slices/N = 6 hearts) and the combination of uncouplers BDM + CytoD (n = 36 slices/N = 4 hearts). Representative long-term force recordings of the first 24 h and 2 h are shown in Figures 1A,B, respectively. In the absence of uncoupling agents all slices exhibited contracture within only a few minutes after the onset of culture, which persisted until the following day. Slices treated with BDM for 15 min exhibited significantly less contracture and many of these slices showed contractions after the washout and 24 h later (Figure 1C). However, also with BDM there were still many slices showing contracture of up to 20 mN (Figure 1D) and no detectable responses to electrical stimulation (Figure 1E), indicated by a missing contraction amplitude. Therefore, we tried other uncouplers, including BTS, blebbistatin and CytoD (Supplementary Figure S1) and several combinations. Although not each possible combination was tested, we found that BDM + CytoD appeared most protective. This was reflected in significantly reduced contracture, which, if present at all, mostly disappeared during the first few hours of culture (Figure 1D), and significantly higher contraction amplitudes after 24 h in culture (Figure 1E).

### 3.2 Survival rates after 24 h and 120 h

In a larger set of 214 slices, we investigated the survival of slices, defined as the presence of a detectable contraction amplitude at 0.5 Hz pacing, after 24 h and 120 h (Table 1). When no uncoupler was used during the initiation phase of the culture, all slices were dead after 24 h (n = 24 slices, N = 3 hearts). When using only BDM as an uncoupler, 72% had measurable amplitudes after day 1, and 59% after day 5. The use of BDM + CytoD during the initiation phase resulted in a survival rate of 90% after day 1% and 76% after day 5. Thus, the relative survival after day 1 in culture until day 5 was 59/72 = 82% in the BDM group and 76/90 = 84% in the BDM + CytoD group, indicating that the addition of CytoD mainly improved the initial survival rate and had no negative effects on long-term survival rate.

**TABLE 1:** Survival rate of slices after 1 day and 5 d in culture when mounted with no (N) uncoupling agent or with 30 mM BDM (B) or with 30 mM BDM +5 µM CytoD (B + C). Survival was defined as the presence of detectable contraction amplitudes in response to 0.5 Hz stimulation.

Time	N (3 animals)	B (6 animals)	B + C (6 animals)
n	24 (100%)	61 (100%)	129 (100%)
1 d	0 (0%)	44 (72%)	116 (90%)
5 d	-	33 (59%)	98 (76%)

### 3.3 Immunofluorescent assessment of cardiomyocyte viability after 24 h

**FIGURE 2:**
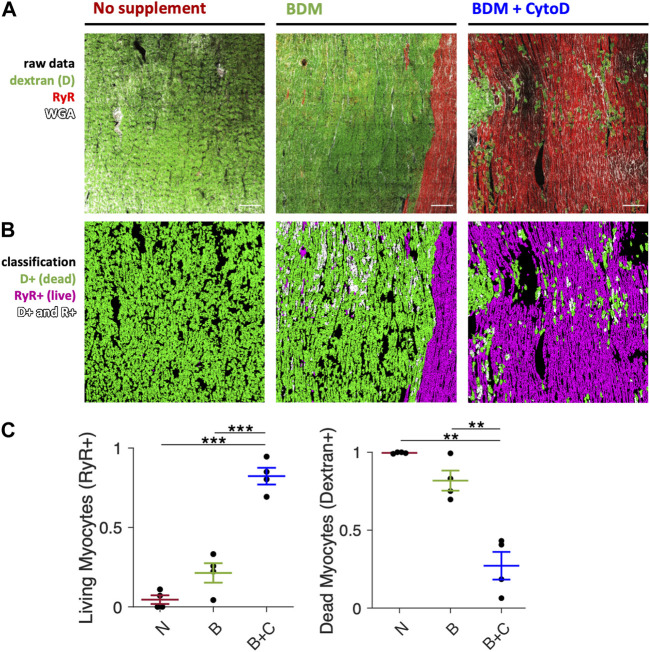
Cardiomyocyte death after initiation of culture. Comparison of slices mounted in culture without electromechanical uncoupling agent, with BDM and BDM + CytoD. Dextran conjugated to FITC as a marker of dead myocytes and immunofluorescence of ryanodine receptor (RyR) as a marker of viable myocytes were applied and visualized with 2D confocal microscopy and quantified. **(A)** Example confocal tile scans (area = 1.6 mm^2^ each) of slices brought into culture without supplement (*left panel*), with 30 mM BDM (*center panel*), or with 30 mM BDM +5 µM CytoD (*right panel*). Slices were stained after 1d of culture with dextran (green), for RyR (red) and extracellular matrix with wheat germ agglutinin (WGA, white). **(B)** Respective segmented and classified images: myocytes stained positive for dextran (D+, green), for RyR (RyR+, red) and positive for both (D+ and R+, white). Scale bar length is 200 µm and applies to all images. **(C)** Quantification of the fraction of RyR-positive (living) and dextran-positive (dead) myocytes in relation to all myocytes in slices mounted without uncoupler (N), with BDM (B) or BDM + CytoD (B + C); n = 4/2 slices/hearts. **p < 0.01, ***p < 0.001, unequal variance, two-tailed t-test with multiple comparison correction.

Based on the functional data shown in Figure 1, where we observed pronounced contracture and absence of stimulated contractions in slices installed in culture without an uncoupling agent or with BDM only, we suspected myocyte injury and necrosis as the cause. Therefore, we assessed the amounts of necrotic cardiomyocytes after 24 h in culture using a live/dead staining assay described recently (Pfeuffer et al., 2023). Figure 2 shows that BDM + CytoD resulted in a significantly higher proportion of living myocytes (82.3% ± 5.3%) and a reduced proportion of myocytes classified as dead (27.2% ± 8.8%) compared to the addition of BDM alone (21.4% ± 6.1% surviving and 81.8% ± 6.5% dead) or no uncouplers at all (4.5% ± 2.7% surviving and 99.6% ± 0.2% dead). Note that the percentages do not exactly add up to 100% because some myocytes are positive for both stainings (Pfeuffer et al., 2023). These results provide evidence of a correlation of contracture with myocyte death on the one hand and contraction amplitude with myocyte survival on the other.

### 3.4 Correlation of contraction amplitude with slice viability

**FIGURE 3:**
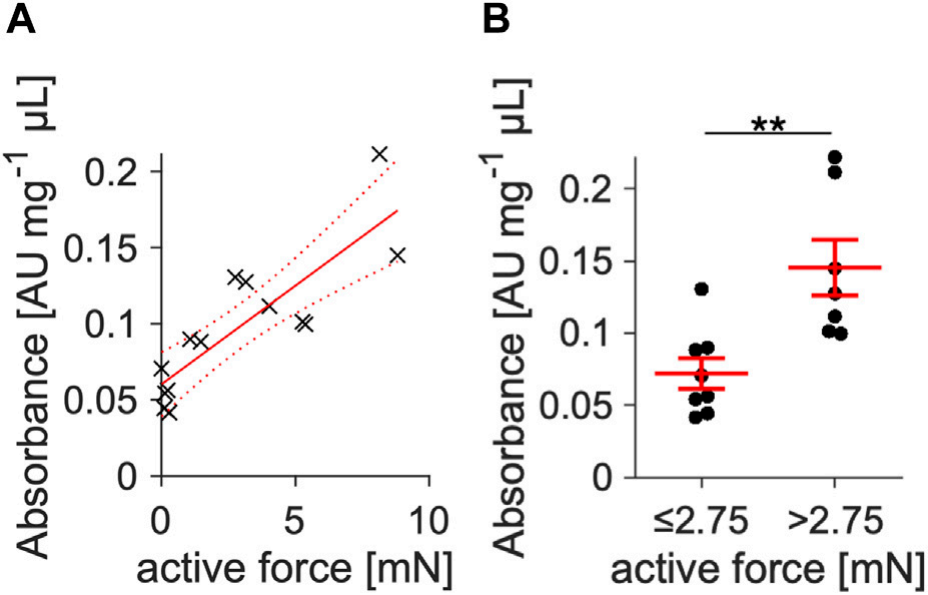
Correlation of contraction amplitude with slice viability. **(A)** Viability was quantified *via* an MTT viability assay in slices kept in culture between 2 and 7 d. All slices were treated with BDM + CytoD during the initial 15–20 min of culture. Absorbance was normalized to total protein content and plotted against contraction amplitude. Linear regression yielded a positive correlation with *p* < 0.001 vs. a constant model and R^2^ = 0.75 (n = 15). **(B)** Using the amplitude median as threshold, slices were divided into a group with low (≤2.75 mN, n = 8) and high (>2.75 mN, n = 7) contracting amplitude. Slices with high amplitude showed significantly higher viability measures.

The results of Figures 1, 2 and a previous study (Pfeuffer et al., 2023) suggest a correlation of contraction amplitude with myocyte viability in the cultured slices. To further investigate this correlation in cultured slices with a different viability test, we conducted an MTT assay on 15 slices that were kept in culture between 2 and 7 days (Figure 3). A linear regression analysis revealed a strong positive correlation of contraction amplitude with MTT absorbance (*p* < 0.001; R^2^ = 0.75; Figure 3A). We further elaborated this relationship by categorizing the slices into two groups using the median amplitude of 2.75 mN as a threshold (Figure 3B). Slices with a high contraction amplitude of >2.75 mN had a significantly higher MTT absorbance than slices with a low contraction amplitude of <2.75 mN. This suggests a reliable correlation between contraction amplitude and tissue slice viability.

Given the results from these sets of experiments, we concluded that the combination of BDM and CytoD effectively reduces initial contracture, improves viability and results in slices with an active contraction amplitude in the range of 1–10 mN. Therefore, in all subsequently described experiments we used BDM + CytoD during the first 15–20 min of culture, followed by a thorough washout. In the next set of experiments, we investigated whether medium additives or different pacing conditions could improve slice function or stability, using contraction parameters as the main indicator. Medium additives or different pacing protocols were not tested in slices mounted without BDM + CytoD.

### 3.5 Improving long-term stability of rabbit ventricular slice culture

#### 3.5.1 Effects of cortisol

**FIGURE 4:**
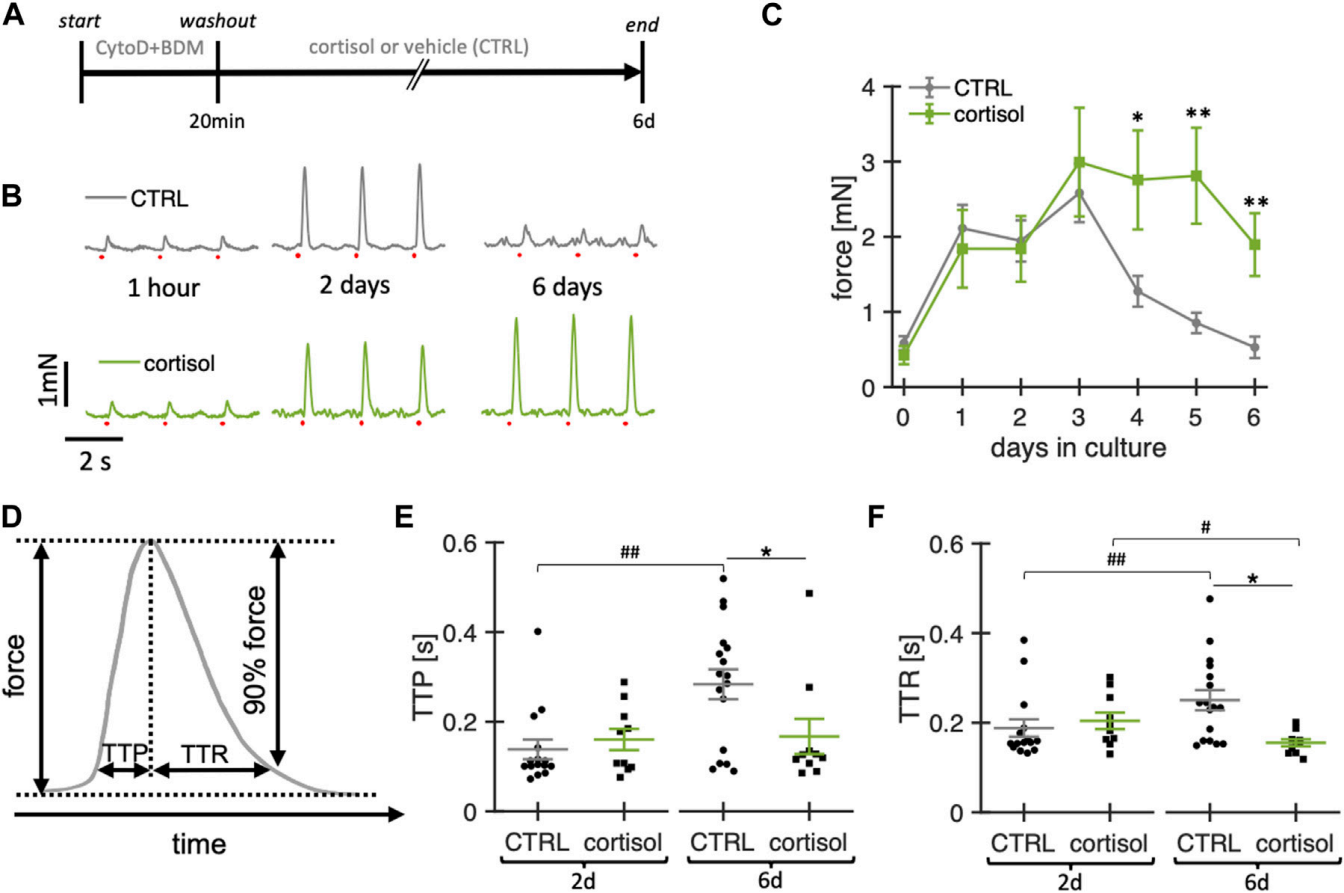
Effect of cortisol on functional stability of rabbit ventricular slices in organotypic culture. **(A)** Timeline (schematic) indicating the times of the experimental interventions. **(B)** example recordings of contractions elicited by field stimulation (red dots) at different time points of a slice cultured under control conditions (CTRL) and with the addition of 20 nM cortisol to the culture medium. **(C)** Summary data of contraction force at 0.5 Hz pacing of CTRL slices (n = 33/10 slices/hearts) and slices treated with cortisol (n = 11/7). **p < 0.01 vs. control, *p < 0.05 vs. control **(D)** Schematic showing how time to peak (TTP) and time to relaxation (TTR) were calculated. **(E, F)** TTP and TTR of CTRL (n = 15/7) and cortisol slices (n = 10/7) at 2d and 6d in culture. Data from matched hearts. *p < 0.05 CTRL vs. cortisol, #*p* < 0.05 2d vs. 6d, ##*p* < 0.01 2d vs. 6d. CTRL vs. cortisol: unequal variance, two-tailed t-test; 2d vs 6d: paired t-test.

Because glucocorticoids are important for stress responses, metabolism and cardiac function (Oakley and Cidlowski, 2015) and have been reported to exert positive effects in cardiomyocyte culture (Seidel et al., 2019), we investigated effects of a physiological concentration of 20 nM cortisol in the culture medium (Figure 4). Figure 4B shows example contraction force recordings of two slices kept in culture for 6 days, one with and one without cortisol in the medium. While at 2 h and 2 d in culture there were no visible changes in contraction amplitude, the cortisol slice appeared stronger than the control after 6 d. Statistical analysis of the contraction force in 11 cortisol-treated slices and 33 control slices confirmed the results of this example (Figure 4C). No differences in amplitudes were detected during the first three days of culture, but after day 4 and onwards, slices not treated with cortisol (control) produced smaller contraction amplitudes, while cortisol-supplemented slices remained almost stable, resulting in significantly larger contraction in the cortisol group when compared with control. Additionally, control slices showed prolongation of time to peak force (TTP) between days 2 and 6, whereas this change in TTP was not detected in cortisol-treated slices (Figure 4E). Given that no significant differences could be detected between cortisol-treated and control slices at day 2 in culture, and that there was no detectable difference between day 2 and day 6 in cortisol-treated slices, we conclude that the addition of cortisol stabilizes the TTP during the 6-day culture. Similarly, time to relaxation (TTR) was increased in control slices after 6 days when compared with day 2 in culture. In cortisol-treated slices, we even observed a decrease in TTR during the same period, albeit with a small effect size (Figure 4F). Taken together, we conclude that the addition of 20 nM cortisol significantly enhances stability and viability, as evidenced by the sustained contraction amplitude. Furthermore, cortisol stabilizes contraction kinetics by preventing the increase in TTP and TTR observed in control cultures. Therefore, we added 20 nM of free cortisol to the culture medium in all subsequent experiments.

#### 3.5.2 Effects of T3 and catecholamines

**FIGURE 5:**
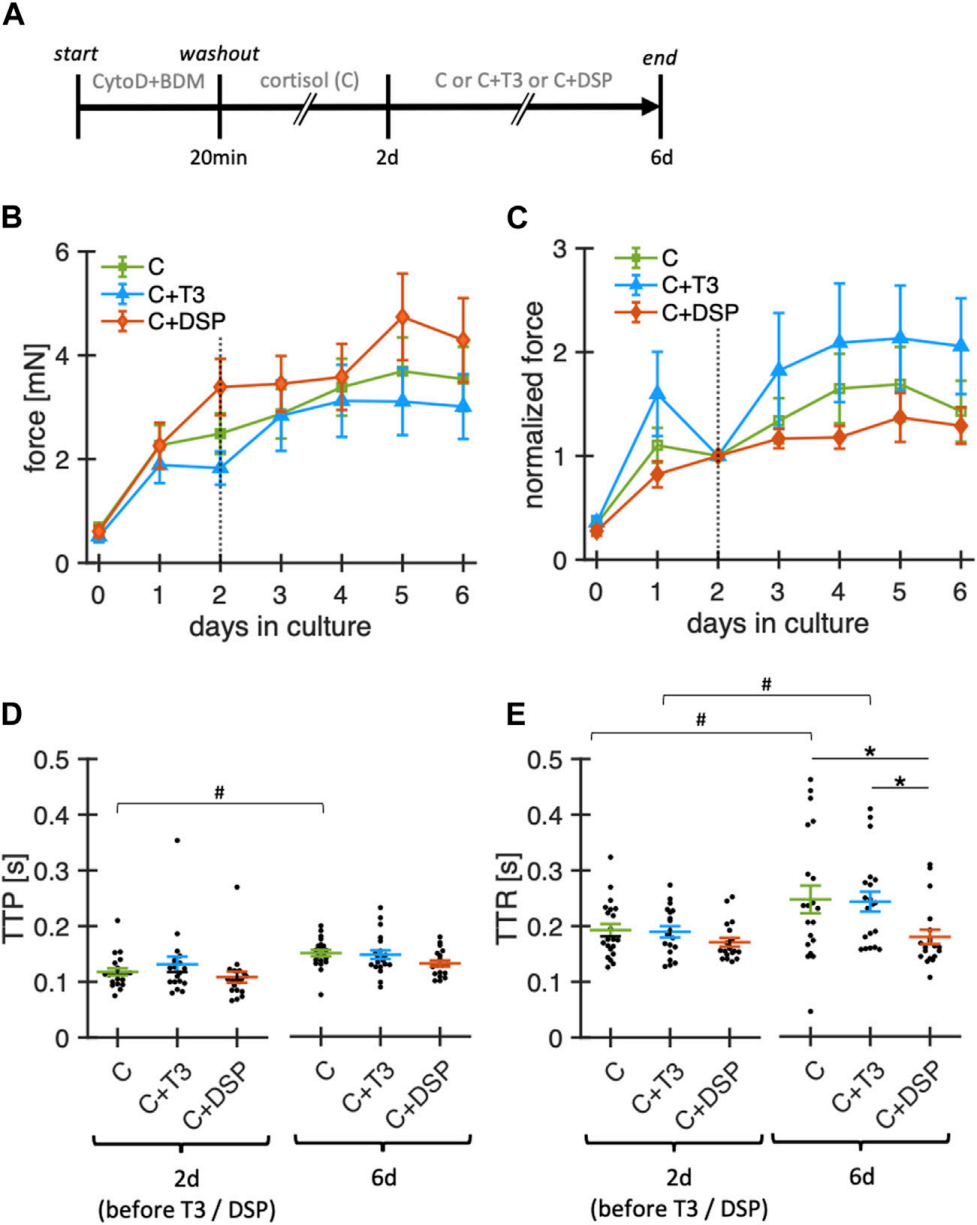
Effect of adrenoceptor stimulation and T3 on functional stability of rabbit ventricular slices in organotypic culture. **(A)** Timeline (schematic) indicating the times of the experimental interventions. **(B)** Summary data of contraction force at 0.5 Hz pacing of slices cultured with 20 nM free cortisol (C, n = 21/7 slices/hearts), cortisol +10 pM fT3 (C + T3, n = 21/7), or 50 nM denopamine, salbutamol and phenylephrine (C + DSP, n = 19/7). **p < 0.01 vs. control, *p < 0.05 vs. control. Note that T3 and DSP were added at day 2. **(C)** Same data as in **(B)**, with the contraction force of each slice normalized to its value at 2 days in culture. **(D, E)** TTP and TTR at 2d and 6d in culture (obtained during 0.5 Hz pacing). Data was retrieved from matched animals, i.e., from each heart, slices were assigned to all groups). *p < 0.05 (unequal variance t-test), #*p* < 0.05 (paired t-test).

Following a similar rationale as for the addition of a physiological concentration of cortisol, we expanded our study to investigate whether baseline stimulation with physiological concentrations of T3 (nominal concentration: 10 pM) and baseline catecholamine stimulation (denopamine 50 nM, salbutamol 50 nM, phenylephrine 50 nM, DSP) in addition to cortisol would be beneficial (Figure 5). Therefore, we cultured ventricular slices with our revised culture conditions as baseline control (20 nM free cortisol, initially with BDM + CytoD) and added T3 or DSP at day 2 in culture. Note that for T3 buffering BSA was added at a concentration of 0.5 mg/mL to the culture medium of all groups. The concentrations of free T3 and free cortisol measured after 24 h of culture were 2.8 ± 0.6 pM (n = 4) and 23.4 ± 0.65 nM (n = 4), respectively. To account for inter-slice heterogeneity, the contraction amplitudes were normalized to their respective amplitudes at day 2.

Over the 6-day period, neither T3 nor the catecholamine cocktail DSP led to significant changes in contraction amplitudes when compared with cortisol alone (Figures 5B,C). Similarly, TTP was not significantly changed by the addition of T3 or DSP. TTR was higher for cortisol-treated (control) and T3-treated slices after 6 days of culture but remained stable when supplemented with DSP. The changes in TTR during culture in the group containing only cortisol are different from those reported in Figure 4. This might be explained by the addition of BSA to the culture medium, which amongst other effects, can bind small amounts of catecholamines (Danon and Sapira, 1972) that can be produced intrinsically in cardiac tissue (Natarajan et al., 2004). A limitation of our experimental design is that we added our mix of adrenoreceptor agonists and T3 only after 2 days in culture. Earlier addition of these substances may still lead to a more successful culture. However, this was done to rule out inter-slice heterogeneities, and provide a basis for the normalization of slices as contraction amplitudes consistently increased during the first 24–48 h in culture. Taken together, stimulation with physiological levels of free T3 or baseline adrenoceptor stimulation with denopamine, salbutamol, and phenylephrine did not detectably increase or further stabilize the contraction amplitude during culture. Denopamine, salbutamol, and phenylephrine, however, might play a role in preserving contraction kinetics.

#### 3.5.3 β-Adrenergic response

**FIGURE 6:**
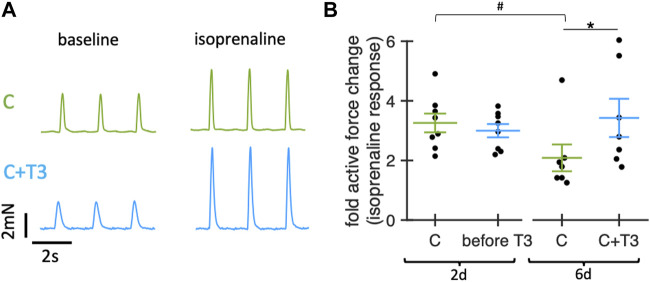
Effect of T3 on beta-adrenergic response of rabbit ventricular slices in organotypic culture. **(A)** Example traces showing contractions of slices cultured for 6d with control medium (20 nM free cortisol, C, green) or with 20 nM free cortisol +10 pM T3 (C + T3, blue) before (baseline) and 5 min after addition of 100 nM isoprenaline. **(B)** Change of active contraction force after addition of 100 nM isoprenaline, normalized to baseline (before addition of isoprenaline) of slices cultured for 2d (before addition of T3) and for 6d (4d after addition of T3). *p < 0.05 vs. C (unequal variance, two-tailed t-test), #*p* < 0.05 6days vs. 2d (paired t-test). N = 16/8 slices/hearts (C) and 15/8 (C + T3). Values obtained from slices of the same animal and group were averaged and presented as one data point. Data was retrieved from matched animals, i.e., from each heart, slices were assigned to all groups).

Because thyroid hormones have been reported to exert stimulating effects on the expression of β-adrenoceptors (Klein, 1988; Bahouth, 1991), we next tested the effect of baseline T3 stimulation on the β-adrenergic response in cultured slices. For this purpose, we added 100 nM isoprenaline and measured the contraction force before and 5 min after the addition (Figure 6). On day 2, before T3 was added, the force increase after isoprenaline stimulation showed no significant difference between the groups. However, on day 6, after 4d of T3 stimulation, slices without T3 supplementation showed a significantly smaller force response post-isoprenaline stimulation. These findings suggest that adding T3 in low physiological concentrations may preserve the β-adrenergic stimulability in long-term culture.

#### 3.5.4 Effects of pacing frequency

**FIGURE 7:**
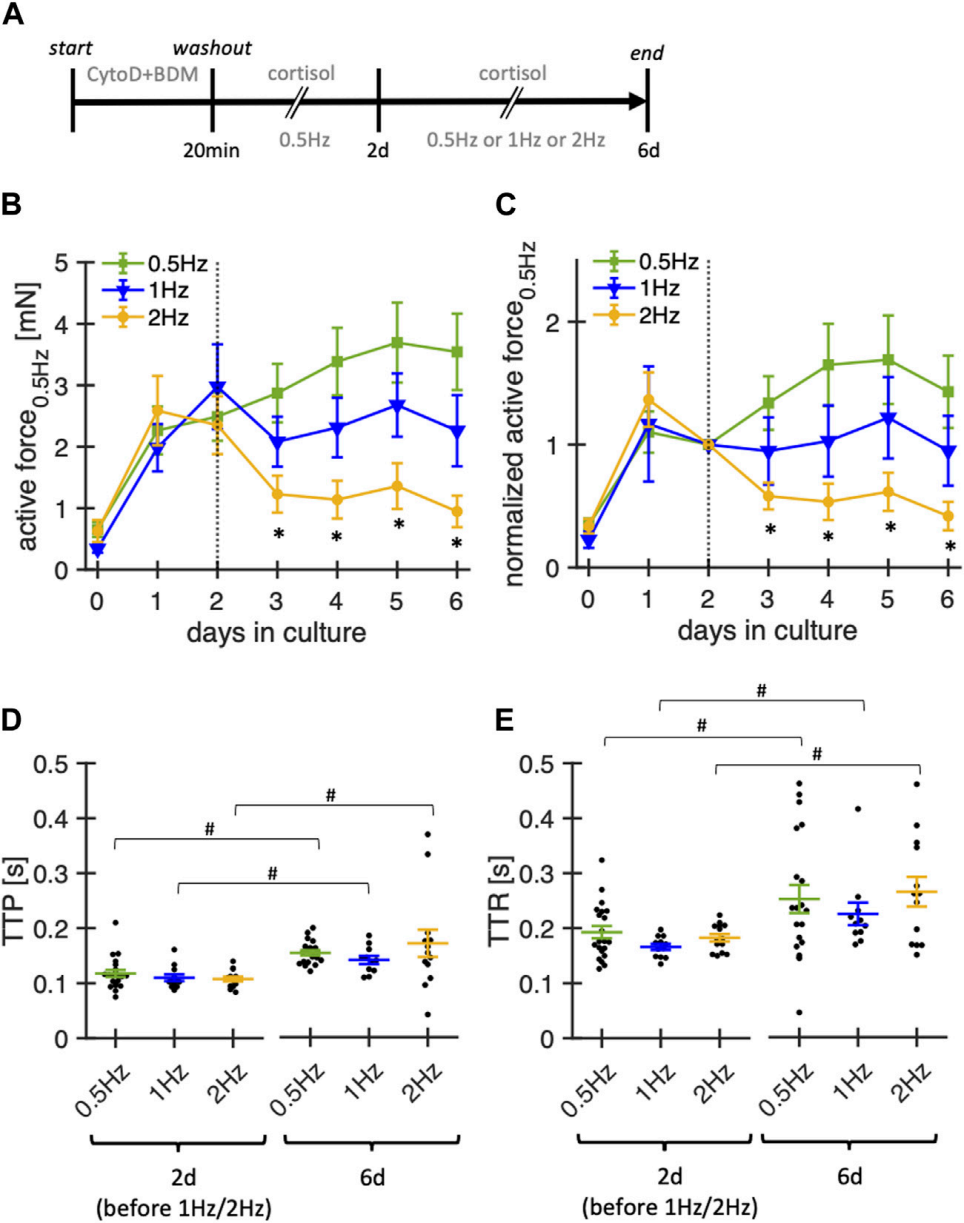
Effect of stimulation frequency on functional stability of rabbit ventricular slices in organotypic culture. **(A)** Timeline (schematic) indicating the times of the experimental interventions. **(B)** Summary data of active contraction force recorded at 0.5 Hz pacing of slices cultured with baseline pacing rates of 0.5Hz, 1 Hz or 2 Hz (n = 21/6, 12/6, 14/6 slices/hearts, respectively). Note that 1 Hz and 2 Hz pacing were started at day 2. During the first 48 h, all slices were paced with 0.5 Hz. Culture medium contained 20 nM free coritsol. *p < 0.05 vs. 0.5 Hz **(C)** Same data as in **(B)**, with the contraction force of each slice normalized to its value at 2 days in culture. **(D, E)** TTP and TTR at 2 d and 6 d in culture (assessed at 0.5 Hz pacing). Data was retrieved from matched animals, i.e., from each heart, slices were assigned to all groups). #*p* < 0.05 (paired t-test 2 d vs 6 d).

In the next experimental series, we investigated the impact of pacing frequencies (0.5 Hz, 1 Hz, 2 Hz) during culture on the stability of contraction amplitude and kinetics in rabbit ventricular slices (Figure 7). Notably, our standard pacing rate of 0.5 Hz is lower than the physiological heart rates of adult rabbits of around 2–3 Hz (Fontes-Sousa et al., 2006). As rabbits are reported to exhibit positive force-frequency relationship, adhering to physiological pacing rates may be beneficial. While we permanently stimulated the slices with the modified pacing rates, the reported amplitudes were measured at 0.5 Hz after a short equilibration phase to render the groups comparable by avoiding frequency-dependent short-term effects. During the first 2 d, all slices were subjected to control conditions (0.5 Hz pacing rate, 20 nM free cortisol). On day 2, the slices were randomly assigned to the three different pacing frequencies.

Significantly lower amplitudes were observed for slices paced at 2 Hz, beginning from day 3 in culture, that is, already 1 day after switching to 2 Hz pacing. TTP increased along all three pacing conditions when comparing measurements from day 2 to day 6. Similarly, TTR increased in all conditions over the same time. Although not tested systematically, we also paced some slices with only 0.2 Hz, which, however, did not improve the stability (data not shown). Based on these findings, pacing rates of 0.5–1 Hz seem to provide the most stable conditions for rabbit ventricular slices with the culture system used here.

### 3.6 Metabolic effects of pacing frequency and T3

**FIGURE 8:**
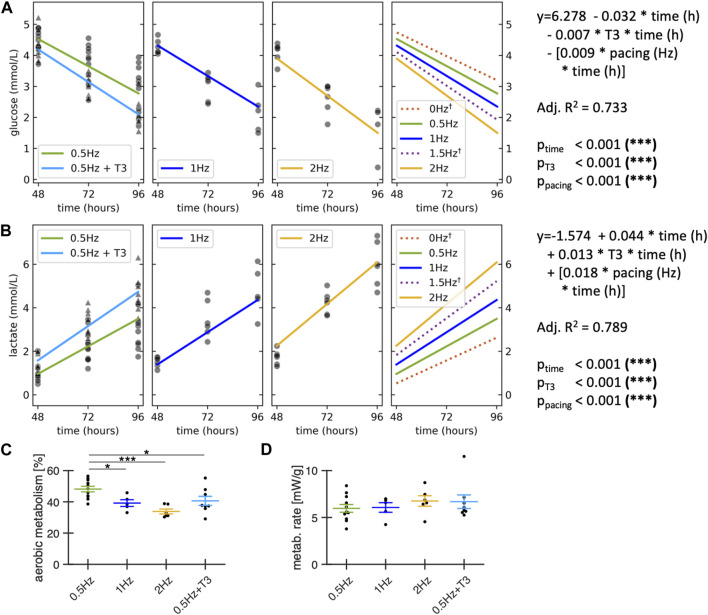
Metabolic effects of stimulation frequency and T3 supplementation. All groups contained 20 nM cortisol. Control and T3 were paced with 0.5 Hz. Glucose **(A)** and lactate concentration **(B)** were measured in the medium immediately after the first medium exchange at 48 h in culture, at 72h and 96h. A linear regression analysis of glucose and lactate concentration was performed across all data points (n = 92 measurements; N = 31 slices; M = 7 rabbits), modelled as a function of time with interaction terms for T3 supplementation and pacing frequencies. †: Regression lines for 0 Hz and 1.5 Hz are model predictions for visualization purposes, no datapoints were collected at 0 and 1.5 Hz. **(C)** Estimated contribution of aerobic metabolism to ATP production. **(D)** Estimated metabolic rate during the first 48h after medium exchange. Data obtained from matched animals. *p < 0.05, **p < 0.01, ***p < 0.001 **(A, B)** equal variance, two-tailed t-test; **(C, D)**: unequal variance, two-tailed t-test.

Because culturing slices at pacing rates of 2 Hz had a significantly negative effect on slice contraction amplitudes, we suspected increased metabolism or insufficient oxygen supply as possible causes. We therefore explored the effect of pacing rates and T3 supplementation on glucose consumption and lactate production during culture in a total of 31 slices from 7 rabbit hearts (Figure 8). Absolute glucose levels in the media were quantified at the following time points: immediately after partial medium exchange (0 h), 24 h post-medium change, and 48 h post-medium change. Our analysis revealed that a pacing rate of 2 Hz as well as T3 supplementation increased glucose consumption by approx. 50% and 20%, respectively (Figure 8A), which was accompanied by a 80% and 30% higher lactate production compared to the control group (Figure 8B). This indicates that large part of the additionally metabolized glucose was used for anaerobic glycolysis. In fact, when estimating the amount of ATP produced by aerobic metabolism, we found a significant reduction *versus* control with higher pacing rates or T3 supplementation (Figure 8C). Although there was a tendency towards higher metabolic rates with T3 or high pacing rate (Figure 8D), we did not find significant differences, which can be explained by the lower ATP yield per glucose molecule in anaerobic glycolysis.

### 3.7 Microstructural volume fractions and TATS density

**FIGURE 9:**
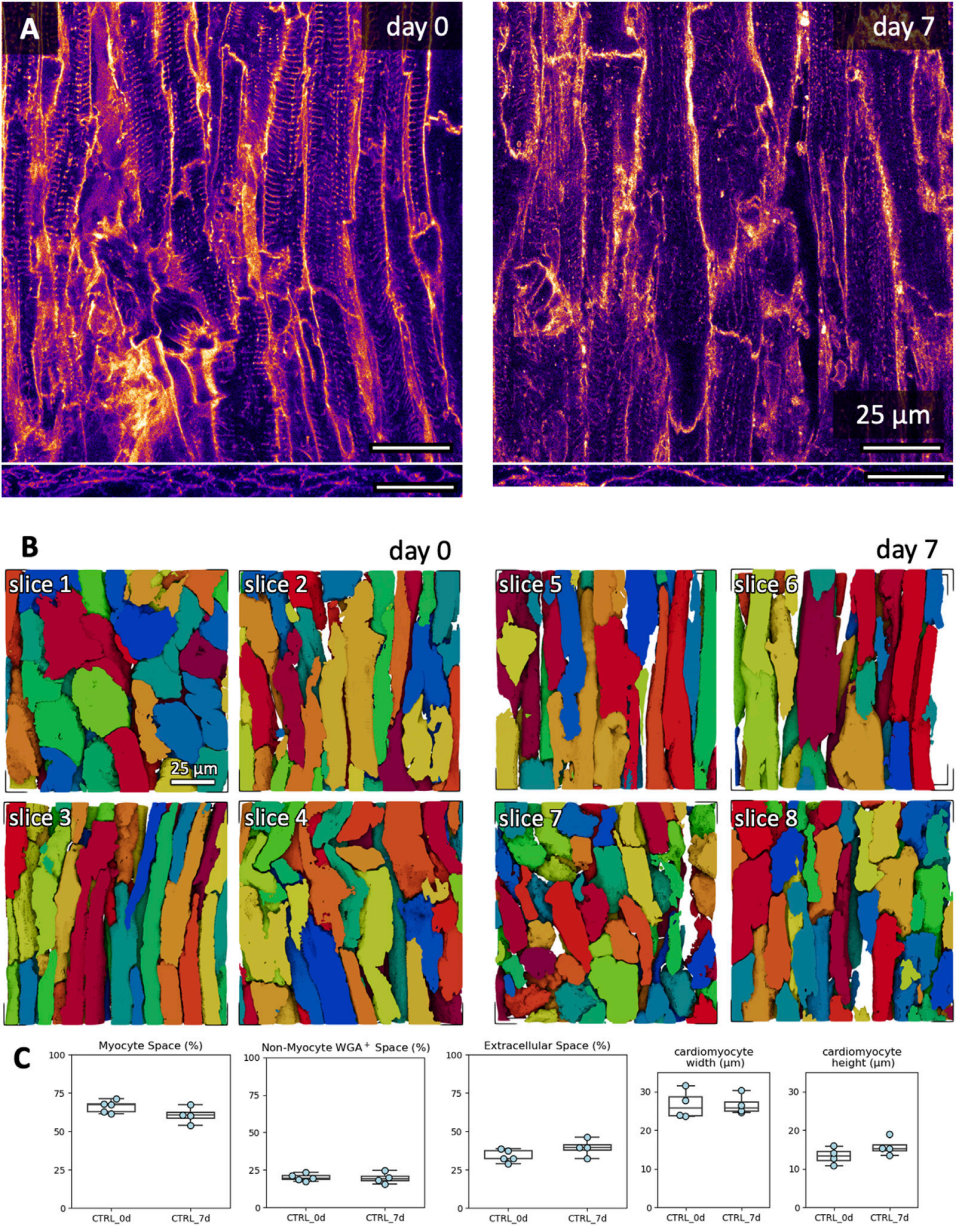
Microstructural assessment of freshly cut and 7-day cultured tissue. Culture conditions included initiation with BDM and cytochalasin D, and medium with 20 nM free cortisol. **(A)** Example XY (top) and XZ (bottom) images of the WGA signal in a 3D confocal image stack in freshly cut and 7-day cultured tissue slices. **(B)** Exemplary 3D reconstructions of individual cardiomyocytes from fresh slices (day 0, slices 1-4) and cultured slices (day 7, slices 5-8). The scale bar in slice one applies also to slices 2-8. **(C)** Quantifications of myocyte space, WGA-positive non-myocyte space (excludes the TATS), extracellular space (includes the TATS) and cardiomyocyte width and height. WGA-positive non-myocyte space is a measure correlating with fibrotic remodelling. Up to three 3D volumes were investigated per tissue slice; the scatter points represent the average value per slice (analyzed 3D volumes: n = 22, analyzed slices: N = 9). One slice was excluded from the width and height analysis due to cardiomyocytes being oriented orthogonally to the imaging plane (stack shown in B, day 0, top left). No significant difference was detected between groups (equal variance, two-tailed t-test).

We assessed changes in microstructure during 7 days of culture using fluorescent staining and 3D confocal microscopy. We selected the combination of BDM + CytoD during mounting in culture and 20 nM of free cortisol as a medium supplement. Examples of the fluorescent signals of a fresh and cultured slice are shown in Figure 9A. Furthermore, we reconstructed individual cardiomyocytes in the tissue stacks based on the structural information provided by the WGA signal (Figure 9B). Cardiomyocytes were mostly oriented in-plane or diagonally to the optical section plane of the confocal microscope, and, thus, the cutting direction. We calculated the volume fractions of cardiomyocytes, WGA-negative and WGA-positive intermyocyte space (often referred to as ‘extracellular space’ from a myocyte-centric view; Figure 9C). There were no significant differences in the calculated volume constituents in freshly cultured tissue slices compared to 7-day-cultured tissue slices. Also, we did not observe any changes in the width or height of reconstructed cardiomyocytes.

**FIGURE 10:**
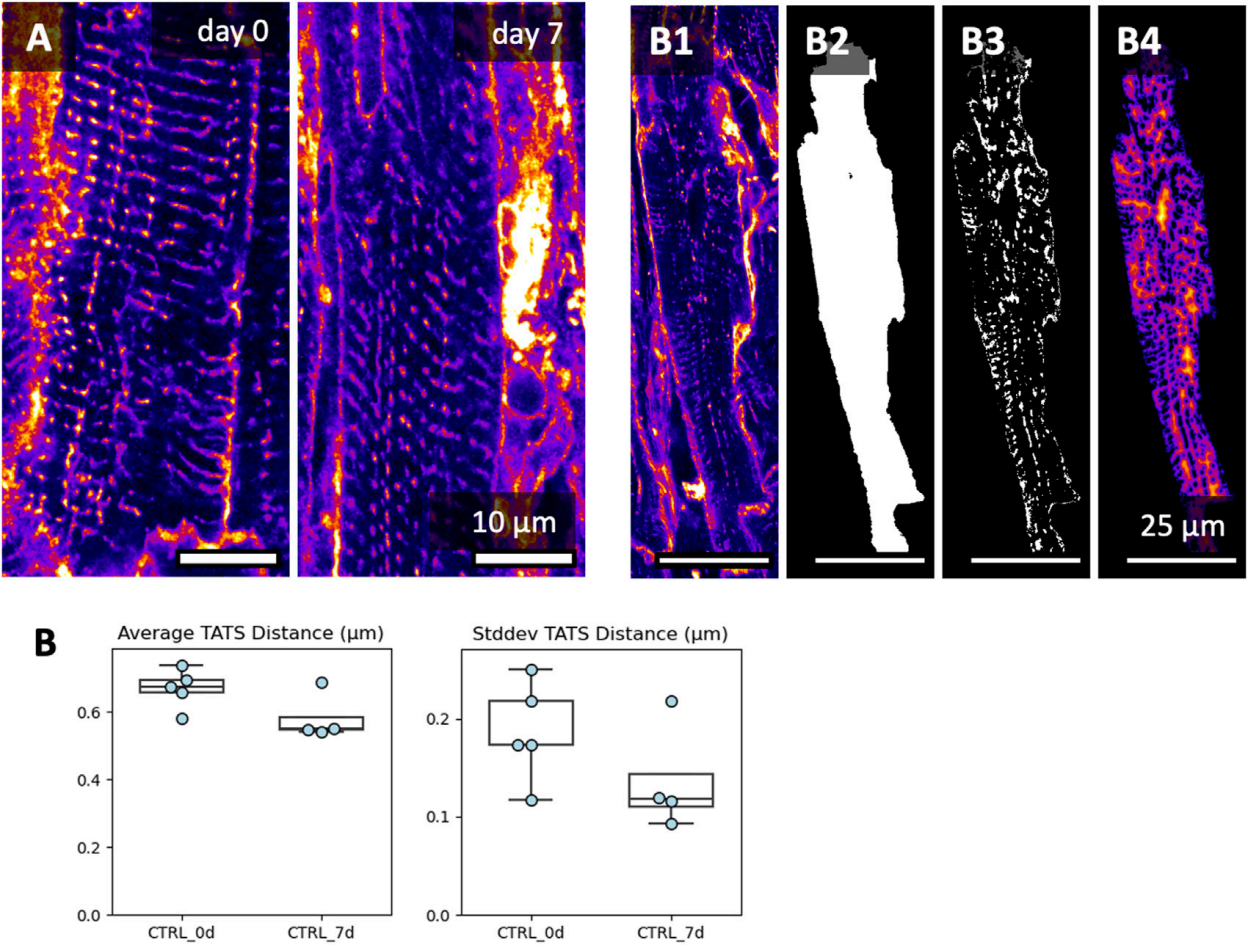
Image-based assessment of the transverse-axial tubular system’s (TATS) density after 7 days of culture. Culture conditions included BDM + CytoD during initiation, and 20 nM cortisol during maintenance. **(A)** XY views and respective zoom-ins of the transverse-axial tubular system at day 0 and day 7. Here, we picked regions with well-preserved TATS, showing that TATS can be preserved for 7 days of culture. Additional image data is shown in Supplementary Data S1. **(B)** To quantify the TATS density, the WGA signal **(B1)**, was masked with each cardiomyocyte mask **(B2)**, thresholded **(B3)**, and then used to calculate the Euclidean 3D distance from the cytoplasm to the TATS **(B4)**. Up to three 3D volumes were investigated per tissue slice; the scatter points represent the average value per slice (analyzed 3D volumes: n = 22, analyzed slices: N = 9). No significant difference was detected between groups (equal variance, two-tailed t-test).

The transverse (-axial) tubular system, TATS, is known to be remodeled during the culture of cardiomyocytes (Hammer et al., 2010). We investigated the TATS in the acquired 3D image volumes in freshly cut and 7-day cultured tissue slices (Figure 10). The glycocalyx, labelled by WGA, reveals dense TATS in freshly and 7-day-long cultured tissue slices (Figure 10A). Occasionally, we observed irregular TATS and cardiomyocytes in contracture in both fresh and 7-day-long cultured slices. Measurements of the 3D distance from the cardiomyocyte cytoplasm to the TATS showed no significant differences over the culture period (Figure 10B). In summary we conclude that the microstructure of tissues and myocytes did not change significantly during 7 d of culture.

### 3.8 Calcium imaging

**FIGURE 11:**
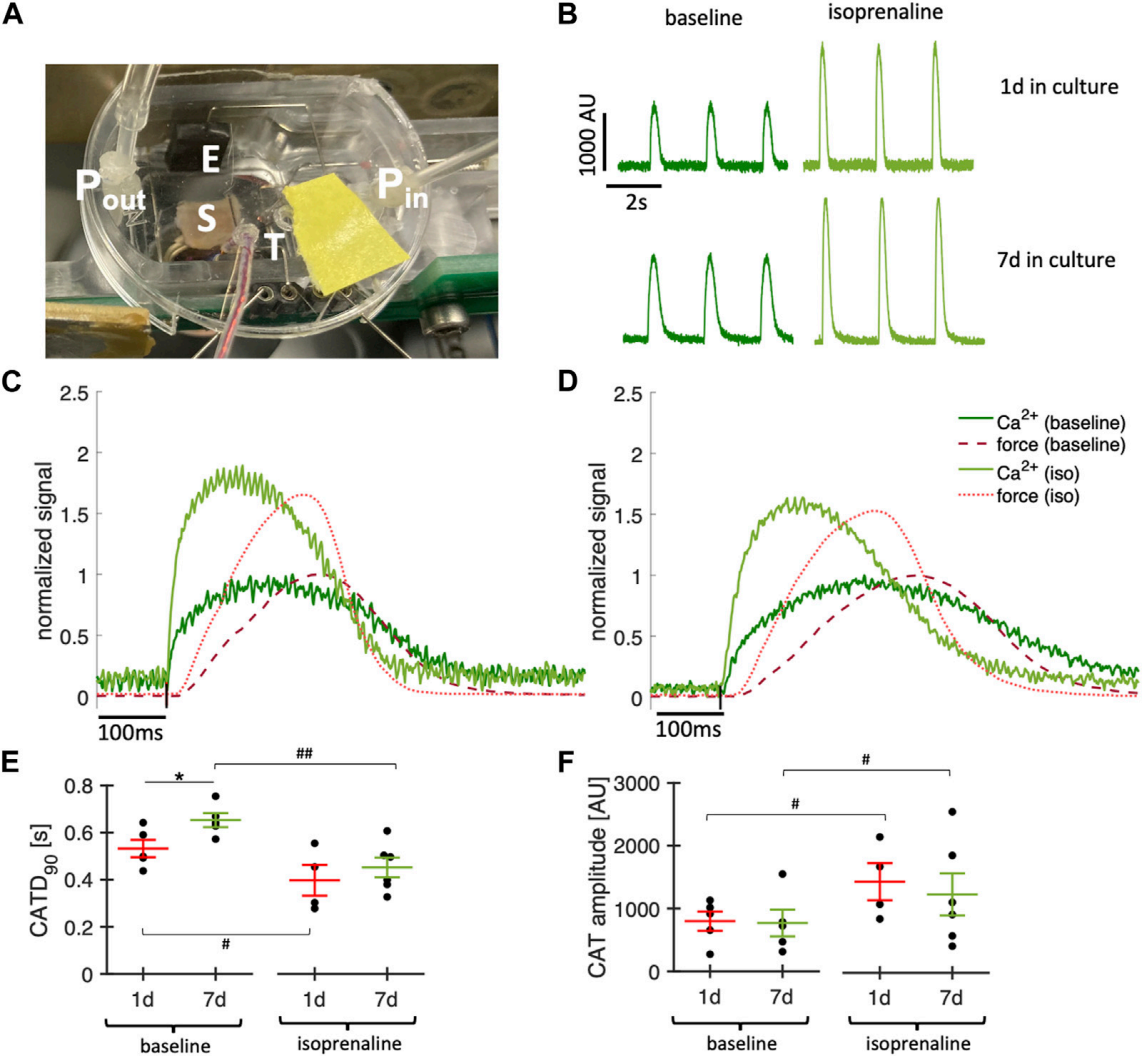
Simultaneous Ca^2+^ imaging and force recordings. **(A)** Photograph showing a culture chamber with mounted tissue slice (S), temperature probe (T), perfusion with inflow (P_in_) and outflow (P_out_) and electrodes for stimulation (E). **(B)** Examples of Ca^2+^ signals recorded in a slice after 1d and a slice after 7d in culture before (baseline) and after addition of 100 nM isoprenaline. **(C)** Overlay of recorded contraction force (red) and Ca^2+^ signal (green) before (dark red, dark green) and after addition of 100 nM isoprenaline (light red, light green) in a slice after 1d in culture. Ca^2+^ signal and force were normalized to the respective maximum at baseline. **(D)** Same as **(C)** in a slice after 7d in culture. **(E)** Ca^2+^ transient duration at 0.5 Hz pacing in slices after 1d and 7d in culture before (baseline) and after addition of 100 nM isoprenaline. **(F)** Ca^2+^ amplitudes measured at 0.5 Hz pacing. *p < 0.05 1d vs. 7d (unequal variance, two-tailed t-test), #*p* < 0.05, ##*p* < 0.01 baseline vs. isoprenaline (paired t-test).

Simultaneous Ca^2+^ imaging and contraction force recording provides valuable insight into excitation-contraction coupling and Ca^2+^ cycling. We demonstrate our approach in Figure 11, showing example Ca^2+^ signals of a slice that was kept in culture for 1 day or 7 days. The overlay of the signals, which were recorded in the absence and presence of the β-adrenergic agonist isoprenaline (100 nM), show the expected increasing effect on the amplitude and kinetics of the Ca^2+^ transient and contraction (Figures 11C,D). To test whether the Ca^2+^ signal changed during culture, we compared 4 slices kept in culture for 1d with 5 slices kept in culture for 7d at a pacing rate of 0.5 Hz and 37°C during the recordings. Ca^2+^ transient duration was increased after 7d of culture (Figure 11E), but still showed a significant response to isoprenaline, which fits to a prolonged contraction observed in cultured slices in the absence of catecholamines (Figure 5). The relatively long Ca^2+^ transient amplitude shortened significantly at higher pacing rates (not shown). The Ca^2+^ transient amplitude, however, was not significantly different in day 1 and day 7, but in both cases increased significantly with isoprenaline stimulation (Figure 11F). In summary, these results illustrate responsiveness to catecholaminergic stimulation of cardiac slices after 1 week in culture and provide evidence for conserved cardiomyocyte Ca^2^^+^ cycling and demonstrate a methodology for comprehensive correlation of Ca^2+^ dynamics with force developments in cardiac slice cultures.

### 3.9 Sharp electrode measurements

**FIGURE 12:**
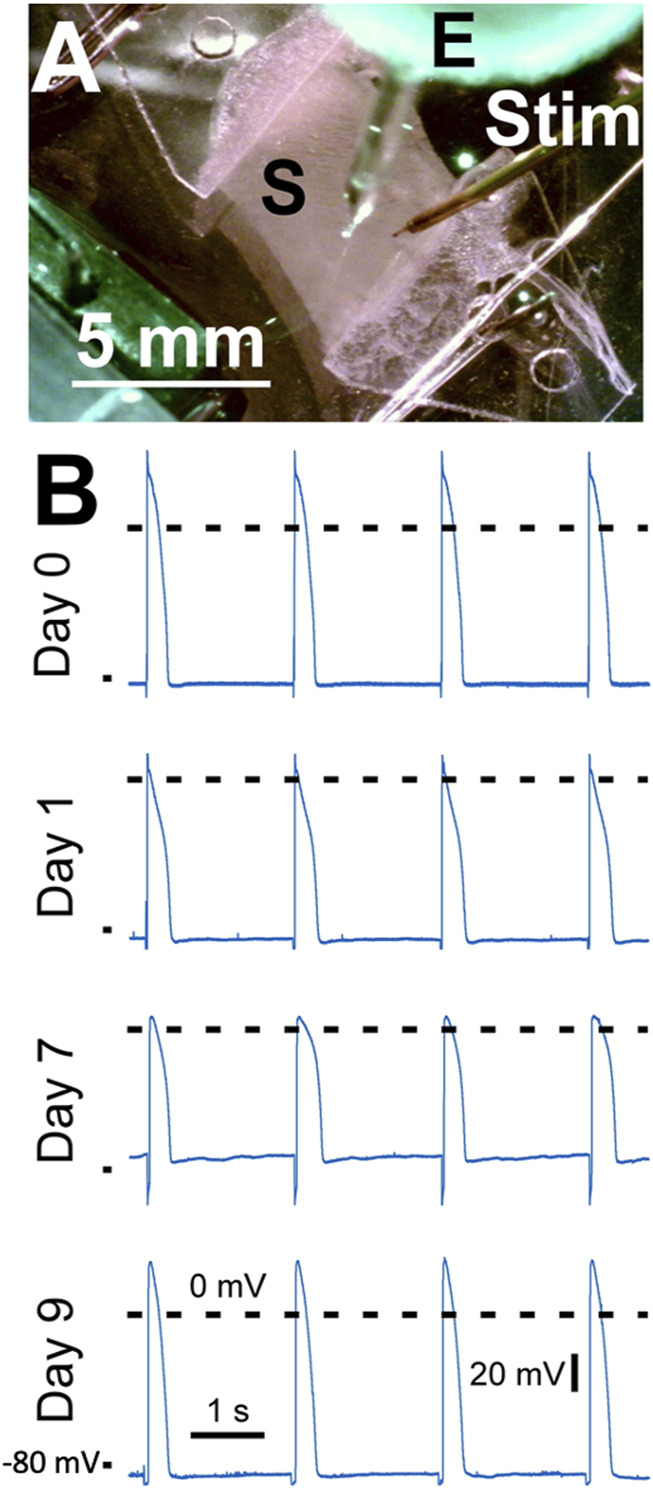
Action potential recordings from left ventricular slices cultured for up to 9 days. **(A)** The slice (S) mounted in a biomimetic chamber was electrically stimulated by a point stimulator (Stim) while a sharp microelectrode (E) was used to record the membrane potential. **(B)** Action potential recordings obtained at 37°C at different times in culture. Day 0 corresponds to the day of heart excision. Dashed lines show 0 mV, the mark on the bottom left of each recording indicates −80 mV. Pacing frequency was 0.5 Hz. The stimulation artefact was removed by filtering.

In Figure 12 we demonstrate sharp electrode measurements in contracting slices kept within the culture chambers. Action potentials were recorded in four cultured slices, one slice per culture time points (day 0, day 1, day 7, and day 9; Figure 12B). All recordings showed a stable resting membrane potential and an action potential typical for rabbit ventricular myocytes paced at 0.5 Hz (Harada et al., 2011). Calculated parameters of the shown action potentials at days 0, 2, 7 and 9 were as follows: RMP −82 mV, −85 mV, −72 mV, −84 mV, APD90 278 ms, 310 ms, 183 ms, 272 ms, respectively. Further parameters are provided in Supplementary Table S1. Please note that action potential measurements were performed as a proof-of-concept study with n = 1 for each time in culture.

## 4 Discussion

Organotypic culture of cardiac slices is a novel technique, which introduces new challenges regarding the culture conditions required by native cardiac tissue. However, because the throughput with this technique is commonly lower than with cell culture, it is especially challenging to vary culture conditions, such as pacing rates or medium supplements, systematically and conduct enough experiments to allow a solid statistical analysis. Here, we optimized the culture conditions of rabbit ventricular slices with the aim of improving initial survival immediately after placement in culture and long-term stability, using pharmacological or hormonal stimulations that do not cause over-activation of specific signaling pathways. Our main findings include a high susceptibility of rabbit myocardium to sustained contracture and myocyte death when placed in culture, possibly due to rapid temperature changes and reperfusion-like injury. Intriguingly, this problem can be overcome through the use of the electromechanical uncoupling agent and inhibitor of actin polymerization cytochalasin D, making this drug an interesting subject for future studies regarding cardiac ischemia-reperfusion injury. We found that of all stimulants tested, the supplementation of physiological cortisol concentrations had the most pronounced effect on the stability of contraction amplitude and kinetics in long-term culture. Additionally, pacing rate had a profound impact on stability, as well as on metabolic activity. Our results show that rabbit slice culture can be achieved for approximately 7 days with high survival rates, which is sufficient for studies involving gene delivery or long-term pharmacological effects.

### 4.1 Initial damage and electromechanical uncoupling agents

We were surprised that rabbit tissue slices exhibited sustained contracture rapidly and consistently when following protocols published for human cardiac slices and animal slices of different species. The initiation phase of tissue culture comprises re-warming, re-oxygenation, re-initiation of convection, of electrical stimulation and of contraction. Except for the temperature change, all of these events also occur during the reperfusion phase after coronary occlusion. Following this analogy, the cold “ischemic” period when creating tissue slices is at least 2–3 h or, if transport or storage is required, even more than 24 h (Fischer et al., 2019). Considering these similarities, we suggest that the damage observed here or in other studies (Fischer et al., 2019) during the first hours of culture is comparable in many aspects to ischemia-reperfusion injury after coronary occlusion. This initial damage, indicated by sustained contracture, was reduced by the addition of electromechanical uncouplers, with the combination of BDM and CytoD proving particularly effective. The combination of BDM with other uncouplers, however, did not show promising results in an explorative experiment (Supplementary Figure S1). Blocking the actin-myosin interaction by myosin II inhibitors has been shown earlier to protect from cutting injury (Mulieri et al., 1989; Wang et al., 2015). It is possible that BDM and CytoD act synergistically in blocking actin-myosin interactions and therefore prevented contracture and subsequent cell death (Calaghan et al., 2000; Kettlewell et al., 2004). While BDM inhibits myosin II and is also a dephosphorylation agent (Stapleton et al., 1998), CytoD interacts with actin. Thus, combinations of a myosin inhibitor with an actin inhibitor may result in synergistic effects. However, because the concentration of BDM was in the range reported to cause nearly complete electromechanical uncoupling (Biermann et al., 1998), the additional protective effects of CytoD may result from effects on the actin cytoskeleton rather than the sarcomeric actin. In fact, it has been demonstrated that CytoD in the low micromolar range can prevent ischemia-reperfusion injury in liver cells (Shi et al., 2009).

Even though we applied CytoD for only a brief period of 15–20 min and did not observe negative effects on contraction force after successful mounting in culture when compared to BDM alone (Table 1) we cannot fully exclude that the transient myosin inhibition via BDM or actin disruption via CytoD may have any permanent effects on Ca^2+^ handling or electrophysiology in long-term culture. One study reported that the effects on contraction and action potential parameters of BDM were completely reversible after washout, while CytoD effects on contraction partially remained 15 min after the washout. However, the concentration of CytoD was 16 times higher than here (Biermann et al., 1998). In a study using Langendorff-perfused rabbit hearts, action potential duration was still increased by approx. 10% at 1 h after washout of CytoD, while conduction delay, however, was completely reversible (Kettlewell et al., 2004). Yet another study found that 0.5 µM CytoD preserves function and morphology of cultured adult ventricular myocytes (Tian et al., 2012).

One should note that others did not report initial contracture in rabbit slice culture, which could result from a different mode of anesthesia or initial cardioplegic perfusion. However, before rewarming to 37°C, an equilibration period at room temperature in the presence of 30 mM BDM was used, which may have had a similar effect as the equilibration period used here at 37°C (Watson et al., 2019). An earlier study conducting acute experiments in rabbit and guinea pig cardiac slices at 37°C also described rewarming of the slices in BDM- or blebbistatin-containing medium (Wang et al., 2015). Overall, this suggests a particularly high susceptibility to temperature- or ischemia-reperfusion-related injury in rabbit slices. Furthermore, we suggest to investigate the potentially protective effects of cytochalasin D and other drugs acting on the actin cytoskeleton in ischemia-reperfusion injury. In summary, we achieved a survival rate at culture day 6 of 76%, compared to 59% with BDM only, and 0% with no added uncoupler. Our optimized survival rates are similar to other tissue culture studies for human atrial trabecular (Klumm et al., 2022) and human ventricular myocardium (Fischer et al., 2019).

### 4.2 Cortisol

Cortisol at physiological levels was very effective in stabilizing slice function, especially after 2–3 days in culture. This suggests that cortisol-induced changes in gene expression are responsible for the improved stability and fits to studies by us and others that show glucocorticoid signaling is important for cardiac function (Oakley et al., 2013; Rog-Zielinska et al., 2013; Oakley and Cidlowski, 2015). It would be interesting to investigate if higher concentrations exert additional beneficial effects on slice stability and function and if the effect also applied to myocardial slices from other species. However, our goal was to test physiological concentrations, providing conditions that resemble those found *in vivo*. In fact, the lack of cortisol in the medium can be considered non-physiological. We therefore recommend the addition of cortisol in free concentrations of 20–50 nM.

### 4.3 T3, catecholamines

Another important hormone that has been described to be important for heart function and development as well as myocyte structure and function is the thyroid hormone T3 (Grais and Sowers, 2014; Parikh et al., 2017; Jessica et al., 2022). We found that low concentrations of fT3 (nominal: 10 pM, measured: 3–5 pM) had no significant effects on contraction amplitudes, but improved β-adrenergic response and increased the metabolism as indicated by elevated glucose consumption. This fits to the known effects of T3 on the expression of the β_1_ receptor in the heart. Thyroid hormones have been reported to exert stimulating effects on the expression of β-adrenoceptors (Klein, 1988; Bahouth, 1991) and on metabolism in general. Our findings also show that the albumin buffering and respective calculations we applied were reasonable estimations of the actual free T3 concentration. Other studies using T3 usually did not consider or apply buffering by albumin or other proteins and used concentrations of T3 up 100,00× higher than the physiological levels of free T3 (Parikh et al., 2017; Jessica et al., 2022; Pitoulis et al., 2022). Here we demonstrated that concentrations of free T3 in the low picomolar range exert positive effects on the slices and increase metabolism, which we would therefore recommend. Monitoring of free T3 24 h after medium change showed a lower concentration than calculated. This could be due to degradation during culture or to a higher actual affinity constant of BSA than that reported for human albumin, which was used for the calculation (Yabu et al., 1987). However, free T3 plasma levels in the rabbit were reported to be 3–7 pM (Herrmann et al., 1975; Mustafa et al., 2008), which is still close to the measured concentrations (2.8 pM). It is possible that higher concentrations might further improve slice function but could also accelerate metabolism to a point where oxygen supply becomes insufficient or frequent medium exchange will be required to account for rapid glucose depletion and lactate accumulation.

The addition of a low-concentration catecholamine mixture (DSP) had marginal effects. We found a faster relaxation speed when compared with slices cultured without catecholamines, suggesting an effect on SERCA activity, as expected from β_1_-receptor stimulation. However, otherwise our results do not support the addition of low catecholamine concentrations. Future studies might investigate the combination of baseline catecholamine receptor stimulation in conjunction with T3, considering possible synergistic effects.

### 4.4 Pacing frequency

The physiological resting heart rate of New White Zealand rabbits is 2–3 Hz or 120–180 bpm (Fontes-Sousa et al., 2006). Our initial protocol, adapted from human ventricular slice culture, uses a ∼5x lower pacing rate of 0.5 Hz, which might yield unphysiological effects in rabbit tissues. Therefore, we explored pacing rates of 0.5, 1, and 2 Hz in our study. However, we did not find evidence for improved function in the measured contraction amplitude, TTP or TTR. In contrast, 2 Hz pacing was associated with a significant reduction in slice contraction amplitude and viability. While other parameters, such as electrophysiological properties, might benefit from higher pacing rates, we do not recommend pacing rates above 1 Hz unless changing other parameters of culture conditions. The elevated metabolic activity under these conditions (Figure 8) may necessitate more frequent medium changes and/or higher oxygen pressures in the incubator’s atmosphere. In our experimental setting, the medium was changed every 48 h. After this time, some slices paced with 2 Hz exhibited glucose concentrations less than 2 mmol/L, which may have been responsible for decreased contractility.

### 4.5 Metabolism

We demonstrate an easy method for the estimation of metabolic rate and aerobic vs. anaerobic metabolism, which is possible because glucose is the only nutrient present in M199 medium. The estimated metabolic rates of 3–9 mW/g (Figure 8D) showed a relatively high variance, but were well in the range reported in studies using more sophisticated methods *in vivo* or in perfused hearts (Gibbs and Loiselle, 2001). It was surprising to see no significant differences in metabolic rate between the groups with different pacing rates, considering that at a pacing rate of 1–2 Hz approx. 75% of total cardiac energy consumption should result from its contractile activity (Gibbs and Loiselle, 2001). However, with higher pacing rates we observed a significant reduction in contractile force, almost canceling out the workload expected to be added by higher pacing rates (Figure 7B). Higher pacing rates increased glucose consumption and lactate production rates, suggesting that a lack of oxygen may be responsible for the reduced contraction forces and decreased stability observed with 2 Hz pacing. Therefore, the maximum metabolic rate could be a limiting factor under the culture conditions and media exchange regime used. However, it is unlikely that an increased glucose consumption and higher lactate production are generally detrimental, because although T3 increased these parameters as well, slices supplemented with T3 appeared very stable (Figure 5). Conclusively, the results imply that both high pacing rates as well as T3 supplementation lead to a faster depletion of glucose in the medium, which may require more frequent medium exchanges and/or higher oxygen levels as done by other groups who added high concentrations of T3 (Pitoulis et al., 2022).

It is worth noting that normal myocardium consumes not only glucose, but also lactate, fatty acids and ketone bodies, which were not present in the used culture medium. Lactate accumulated over time, yielding concentrations of 1–6 mM, which is higher than those found in the plasma. Yet, from the ratio of glucose consumed and lactate produced we estimated that up to 50% of ATP production was achieved by using oxidative phosphorylation. This shows that cultured ventricular rabbit slices use aerobic metabolic pathways, but also indicates that there might be either a lack of oxygen supply or mitochondrial dysfunction, because in the normal heart, metabolism is almost exclusively aerobic (Bertero and Maack, 2018). A limitation of providing glucose as the only nutrient in the culture medium is that this could cause non-physiological effects. Adding fatty acids and ketone bodies could provide conditions better resembling those found *in vivo* and might even lead to more pronounced or different effects of supplements such as cortisol or T3, considering their known effects on fatty acid metabolism (Pucci et al., 2000; Macfarlane et al., 2008). However, adding additional nutrients would render it more difficult to assess the metabolic rate.

### 4.6 Microstructure

Maintaining the structural integrity of cultured tissue is key for maximizing the translational value of the culture. Within several days of culture, isolated cardiomyocytes undergo remodeling processes, including their morphology (Mitcheson et al., 1996; Hammer et al., 2010) and TATS (Lipp et al., 1996; Louch et al., 2004). Therefore, changes in myocyte volume fractions, morphological measures, and TATS density were investigated. Our findings revealed no significant differences in these parameters after 7 days of culture. Additionally, the observed volume fractions are consistent with our previous study on perfusion-fixed ventricular rabbit tissue (Greiner et al., 2018). Concerning structural integrity, we also considered potential fibroblast activation and fibrosis. To this end, we used WGA labeling, a suitable marker for quantifying fibrotic tissue constituents (Emde et al., 2014). Our results showed no increase in WGA-labeled intermyocyte space, which would be expected after fibroblast activation or fibrosis. These preserved structural features are key advantages of biomimetic cultures with mechanical preload compared to the culture of singular cells and show that rabbit myocardial slice culture can be used for both functional and structural long-term studies *in vitro*.

### 4.7 Conclusions

This study shows that rabbit hearts, which have been used for many decades in cardiovascular research due to similarities with human cardiac electrophysiology, excitation-contraction coupling and microstructure, can be used for myocardial slice culture lasting up to 7 days. We suggest that this will enable cost-effective *in-vitro* experiments to obtain new insights into cardiac physiology and pathophysiology. For example, one could investigate mechanisms of fibrosis and other types of tissue remodeling, screen for cardiotoxicity of newly developed pharmacological agents or edit genes in highly preserved functional myocardium. These and other conceivable studies can be carried out without additional suffering for the animals and without the need for authorization, as they are performed *in vitro*. However, our study also shows that the behavior of rabbit heart slices during the initiation of culture differs from that of human cardiac slices or slices from hearts obtained from other species, suggesting differences in metabolism, the response to temperature changes or to ischemia-reperfusion in general. In this respect, rabbit myocardium may be a less suitable model. Furthermore, while our study demonstrates stability over the course of 1 week, we propose to address in future studies if and how long-term stability over several months in culture as demonstrated in human cardiac slices can be achieved in animal slices.

## Data Availability

The raw data supporting the conclusion of this article will be made available by the authors, without undue reservation.

## References

[B1] Abd-Allah N. M. Hassan F. H. Esmat A. Y. Hammad S. A. (2004). Age dependence of the levels of plasma norepinephrine, aldosterone, renin activity and urinary vanillylmandelic acid in normal and essential hypertensives. Biol. Res. 37 (1), 95–106. 10.4067/s0716-97602004000100010 15174309

[B2] Abu-Khousa M. Fiegle D. J. Sommer S. T. Minabari G. Milting H. Heim C. (2020). The degree of t-system remodeling predicts negative force-frequency relationship and prolonged relaxation time in failing human myocardium. Front. Physiology 11 (182), 182. 10.3389/fphys.2020.00182 PMC7083140 32231589

[B3] Amesz J. H. de Groot N. M. S. Langmuur S. J. J. Azzouzi H. E. Tiggeloven V. P. C. van Rooij M. (2023). Biomimetic cultivation of atrial tissue slices as novel platform for *in-vitro* atrial arrhythmia studies. Sci. Rep. 13 (1), 3648. 10.1038/s41598-023-30688-8 36871094 PMC9985600

[B4] Anakwe O. O. Moger W. H. (1984). β2-adrenergic stimulation of androgen production by cultured mouse testicular interstitial cells. Life Sci. 35 (20), 2041–2047. 10.1016/0024-3205(84)90561-7 6149443

[B5] Bahouth S. W. (1991). Thyroid hormones transcriptionally regulate the beta 1-adrenergic receptor gene in cultured ventricular myocytes. J. Biol. Chem. 266 (24), 15863–15869. 10.1016/s0021-9258(18)98488-7 1651924

[B6] Banyasz T. Lozinskiy I. Payne C. E. Edelmann S. Norton B. Chen B. (2008). Transformation of adult rat cardiac myocytes in primary culture. Exp. Physiol. 93 (3), 370–382. 10.1113/expphysiol.2007.040659 18156167

[B7] Bertero E. Maack C. (2018). Metabolic remodelling in heart failure. Nat. Rev. Cardiol. 15 (8), 457–470. 10.1038/s41569-018-0044-6 29915254

[B8] Biermann M. Rubart M. Moreno A. Wu J. Josiah-Durant A. Zipes D. P. (1998). Differential effects of cytochalasin D and 2,3 butanedione monoxime on isometric twitch force and transmembrane action potential in isolated ventricular muscle: implications for optical measurements of cardiac repolarization. J. Cardiovasc Electrophysiol. 9 (12), 1348–1377. 10.1111/j.1540-8167.1998.tb00110.x 9869534

[B9] Brandenburger M. Wenzel J. Bogdan R. Richardt D. Nguemo F. Reppel M. (2012). Organotypic slice culture from human adult ventricular myocardium. Cardiovasc Res. 93 (1), 50–59. 10.1093/cvr/cvr259 21972180

[B10] Calaghan S. C. White E. Bedut S. Le Guennec J. Y. (2000). Cytochalasin D reduces Ca2+ sensitivity and maximum tension via interactions with myofilaments in skinned rat cardiac myocytes. J. Physiol. 2 (Pt 2), 405–411. 10.1111/j.1469-7793.2000.00405.x PMC2270202 11101650

[B11] Danon A. Sapira J. D. (1972). Binding of catecholamines to human serum albumin. J. Pharmacol. Exp. Ther. 182 (2), 295–302. 5048366

[B12] de Boer T. P. Camelliti P. Ravens U. Kohl P. (2009). Myocardial tissue slices: organotypic pseudo-2D models for cardiac research and development. Future Cardiol. 5 (5), 425–430. 10.2217/fca.09.32 19715406

[B13] Dichtel L. E. Schorr M. Loures de Assis C. Rao E. M. Sims J. K. Corey K. E. (2019). Plasma free cortisol in states of normal and altered binding globulins: implications for adrenal insufficiency diagnosis. J. Clin. Endocrinol. Metab. 104 (10), 4827–4836. 10.1210/jc.2019-00022 31009049 PMC6735741

[B14] Emde B. Heinen A. Godecke A. Bottermann K. (2014). Wheat germ agglutinin staining as a suitable method for detection and quantification of fibrosis in cardiac tissue after myocardial infarction. Eur. J. Histochem 58 (4), 2448. 10.4081/ejh.2014.2448 25578975 PMC4289847

[B15] Fischer C. Milting H. Fein E. Reiser E. Lu K. Seidel T. (2019). Long-term functional and structural preservation of precision-cut human myocardium under continuous electromechanical stimulation *in vitro* . Nat. Commun. 10 (1), 117. 10.1038/s41467-018-08003-1 30631059 PMC6328583

[B16] Fontes-Sousa A. P. Bras-Silva C. Moura C. Areias J. C. Leite-Moreira A. F. (2006). M-mode and Doppler echocardiographic reference values for male New Zealand white rabbits. Am. J. Vet. Res. 67 (10), 1725–1729. 10.2460/ajvr.67.10.1725 17014323

[B17] Gibbs C. L. Loiselle D. S. (2001). Cardiac basal metabolism. Jpn. J. Physiol. 51 (4), 399–426. 10.2170/jjphysiol.51.399 11564278

[B18] Goldstein D. S. Eisenhofer G. Kopin I. J. (2003). Sources and significance of plasma levels of catechols and their metabolites in humans. J. Pharmacol. Exp. Ther. 305 (3), 800–811. 10.1124/jpet.103.049270 12649306

[B19] Grais I. M. Sowers J. R. (2014). Thyroid and the heart. Am. J. Med. 127 (8), 691–698. 10.1016/j.amjmed.2014.03.009 24662620 PMC4318631

[B20] Greiner J. Sankarankutty A. C. Seemann G. Seidel T. Sachse F. B. (2018). Confocal microscopy-based estimation of parameters for computational modeling of electrical conduction in the normal and infarcted heart. Front. Physiol. 9, 239. 10.3389/fphys.2018.00239 29670532 PMC5893725

[B21] Greiner J. Sankarankutty A. C. Seidel T. Sachse F. B. (2022). Confocal microscopy-based estimation of intracellular conductivities in myocardium for modeling of the normal and infarcted heart. Comput. Biol. Med. 146, 105579. 10.1016/j.compbiomed.2022.105579 35588677 PMC10195095

[B22] Habeeb A. Sharoud M. N. Basuony H. Michael M. (2018). Effect of environmental climatic conditions on levels of some hormones, vitamins and trace elements in blood and seminal plasma of rabbits. Int. J. Biotechnol. Recent Adv. 1 (1), 18–23. 10.18689/ijbr-1000104

[B23] Hamelberg W. Sprouse J. Mahaffey J. E. Richardson J. A. (1960). Plasma levels of epinephrine and norepinephrine-anesthetic significance. J. Am. Med. Assoc. 172, 1596–1598. 10.1001/jama.1960.03020150020004 14399239

[B24] Hamers J. Sen P. Merkus D. Seidel T. Lu K. Dendorfer A. (2022). Preparation of human myocardial tissue for long-term cultivation. J. Vis. Exp. 184. 10.3791/63964 35723462

[B25] Hammer K. Ruppenthal S. Viero C. Scholz A. Edelmann L. Kaestner L. (2010). Remodelling of Ca2+ handling organelles in adult rat ventricular myocytes during long term culture. J. Mol. Cell Cardiol. 49 (3), 427–437. 10.1016/j.yjmcc.2010.05.010 20540947

[B26] Harada M. Tsuji Y. Ishiguro Y. S. Takanari H. Okuno Y. Inden Y. (2011). Rate-dependent shortening of action potential duration increases ventricular vulnerability in failing rabbit heart. Am. J. Physiology-Heart Circulatory Physiology 300 (2), H565–H573. 10.1152/ajpheart.00209.2010 21148762

[B27] Hengstmann J. H. Goronzy J. (1982). Pharmacokinetics of 3H-phenylephrine in man. Eur. J. Clin. Pharmacol. 21 (4), 335–341. 10.1007/BF00637623 7056280

[B28] Herrmann J. Rusche H. J. Berger M. Kruskemper H. L. (1975). Thyroid function and triiodothyronine and thyroxine kinetics in rabbits immunized with thyroid hormones. Acta Endocrinol. (Copenh) 78 (2), 276–288. 10.1530/acta.0.0780276 803750

[B29] Hjemdahl P. (1993). Plasma catecholamines—analytical challenges and physiological limitations. Bailliere’s Clin. Endocrinol. metabolism 7 (2), 307–353. 10.1016/s0950-351x(05)80179-x 8489483

[B30] Hornyik T. Rieder M. Castiglione A. Major P. Baczko I. Brunner M. (2022). Transgenic rabbit models for cardiac disease research. Br. J. Pharmacol. 179 (5), 938–957. 10.1111/bph.15484 33822374

[B31] Isensee F. Jaeger P. F. Kohl S. A. A. Petersen J. Maier-Hein K. H. (2021). nnU-Net: a self-configuring method for deep learning-based biomedical image segmentation. Nat. Methods 18 (2), 203–211. 10.1038/s41592-020-01008-z 33288961

[B32] Janse M. J. Opthof T. Kléber A. G. (1998). Animal models of cardiac arrhythmias. Cardiovasc. Res. 39 (1), 165–177. 10.1016/s0008-6363(97)00313-1 9764198

[B33] Jessica M. Moustafa M. Qinghui O. Riham A. Xian-Liang T. Abou Bakr S. (2022). Nature portfolio. 10.21203/rs.3.rs-1322108/v1

[B34] Jones C. Robinson R. (1975). Plasma catecholamines in foetal and adult sheep. J. Physiology 248 (1), 15–33. 10.1113/jphysiol.1975.sp010960 PMC1309505 1151803

[B35] Joukar S. (2021). A comparative review on heart ion channels, action potentials and electrocardiogram in rodents and human: extrapolation of experimental insights to clinic. Laboratory Animal Res. 37 (1), 25. 10.1186/s42826-021-00102-3 PMC8424989 34496976

[B36] Kettlewell S. Walker N. L. Cobbe S. M. Burton F. L. Smith G. L. (2004). The electrophysiological and mechanical effects of 2,3-butane-dione monoxime and cytochalasin-D in the Langendorff perfused rabbit heart. Exp. Physiol. 89 (2), 163–172. 10.1113/expphysiol.2003.026732 15123545

[B37] Kino M. Hirota Y. Yamamoto S. Sawada K. Moriguchi M. Kotaka M. (1983). Cardiovascular effects of a newly synthesized cardiotonic agent (TA-064) on normal and diseased hearts. Am. J. Cardiol. 51 (5), 802–810. 10.1016/s0002-9149(83)80137-4 6829441

[B38] Klein I. (1988). Thyroxine-induced cardiac hypertrophy: time course of development and inhibition by propranolol. Endocrinology 123 (1), 203–210. 10.1210/endo-123-1-203 2968237

[B39] Klumm M. J. Heim C. Fiegle D. J. Weyand M. Volk T. Seidel T. (2022). Long-term cultivation of human atrial myocardium. Front. Physiol. 13, 839139. 10.3389/fphys.2022.839139 35283779 PMC8905341

[B40] Lipp P. Huser J. Pott L. Niggli E. (1996). Spatially non-uniform Ca2+ signals induced by the reduction of transverse tubules in citrate-loaded Guinea-pig ventricular myocytes in culture. J. Physiol. 497 (Pt 3), 589–597. 10.1113/jphysiol.1996.sp021792 9003546 PMC1160957

[B41] Louch W. E. Bito V. Heinzel F. R. Macianskiene R. Vanhaecke J. Flameng W. (2004). Reduced synchrony of Ca2+ release with loss of T-tubules-a comparison to Ca2+ release in human failing cardiomyocytes. Cardiovasc Res. 62 (1), 63–73. 10.1016/j.cardiores.2003.12.031 15023553

[B42] Macfarlane D. P. Forbes S. Walker B. R. (2008). Glucocorticoids and fatty acid metabolism in humans: fuelling fat redistribution in the metabolic syndrome. J. Endocrinol. 197 (2), 189–204. 10.1677/JOE-08-0054 18434349

[B43] Marwaha R. K. Tandon N. Ganie M. A. Mehan N. Sastry A. Garg M. K. (2013). Reference range of thyroid function (FT3, FT4 and TSH) among Indian adults. Clin. Biochem. 46 (4-5), 341–345. 10.1016/j.clinbiochem.2012.09.021 23041244

[B44] Milani-Nejad N. Janssen P. M. (2014). Small and large animal models in cardiac contraction research: advantages and disadvantages. Pharmacol. Ther. 141 (3), 235–249. 10.1016/j.pharmthera.2013.10.007 24140081 PMC3947198

[B45] Mitcheson J. S. Hancox J. C. Levi A. J. (1996). Action potentials, ion channel currents and transverse tubule density in adult rabbit ventricular myocytes maintained for 6 days in cell culture. Pflugers Arch. 431 (6), 814–827. 10.1007/s004240050073 8927497

[B46] Mohta M. Dubey M. Malhotra R. K. Tyagi A. (2019). Comparison of the potency of phenylephrine and norepinephrine bolus doses used to treat post-spinal hypotension during elective caesarean section. Int. J. Obstet. Anesth. 38, 25–31. 10.1016/j.ijoa.2018.12.002 30685301

[B47] Mokler C. Mathur P. (1968). Influence of calcium and antiarrhythmic drugs on palmitate uptake by rabbit heart slices. J. Pharm. Sci. 57 (8), 1304–1307. 10.1002/jps.2600570805 5677331

[B48] Moretti A. Fonteyne L. Giesert F. Hoppmann P. Meier A. B. Bozoglu T. (2020). Somatic gene editing ameliorates skeletal and cardiac muscle failure in pig and human models of Duchenne muscular dystrophy. Nat. Med. 26 (2), 207–214. 10.1038/s41591-019-0738-2 31988462 PMC7212064

[B49] Mueller U. W. Potter J. M. (1981). Binding of cortisol to human albumin and serum: the effect of protein concentration. Biochem. Pharmacol. 30 (7), 727–733. 10.1016/0006-2952(81)90158-1 7247957

[B50] Mulieri L. A. Hasenfuss G. Ittleman F. Blanchard E. M. Alpert N. R. (1989). Protection of human left ventricular myocardium from cutting injury with 2,3-butanedione monoxime. Circ. Res. 65 (5), 1441–1449. 10.1161/01.res.65.5.1441 2805252

[B51] Mustafa S. Al-Bader M. D. Elgazzar A. H. Alshammeri J. Gopinath S. Essam H. (2008). Effect of hyperthermia on the function of thyroid gland. Eur. J. Appl. Physiol. 103 (3), 285–288. 10.1007/s00421-008-0701-2 18320209

[B52] Nagao T. Nakajima H. (1989). Denopamine. Cardiovasc. Drug Rev. 7 (4), 310–325. 10.1111/j.1527-3466.1989.tb00534.x

[B53] Naito K. Nagao T. Otsuka M. Harigaya S. Nakajima H. (1985). Studies on the affinity and selectivity of denopamine (TA-064), a new cardiotonic agent, for β-adrenergic receptorst. Jpn. J. Pharmacol. 38 (3), 235–241. 10.1254/jjp.38.235 2997524

[B54] Natarajan A. R. Rong Q. Katchman A. N. Ebert S. N. (2004). Intrinsic cardiac catecholamines help maintain beating activity in neonatal rat cardiomyocyte cultures. Pediatr. Res. 56 (3), 411–417. 10.1203/01.PDR.0000136279.80897.4C 15333759

[B55] Nerbonne J. M. (2000). Molecular basis of functional voltage-gated K+ channel diversity in the mammalian myocardium. J. Physiol. 2 (Pt 2), 285–298. 10.1111/j.1469-7793.2000.t01-1-00285.x PMC2269952 10835033

[B56] Oakley R. H. Cidlowski J. A. (2015). Glucocorticoid signaling in the heart: a cardiomyocyte perspective. J. Steroid Biochem. Mol. Biol. 153, 27–34. 10.1016/j.jsbmb.2015.03.009 25804222 PMC4568128

[B57] Oakley R. H. Ren R. Cruz-Topete D. Bird G. S. Myers P. H. Boyle M. C. (2013). Essential role of stress hormone signaling in cardiomyocytes for the prevention of heart disease. Proc. Natl. Acad. Sci. U. S. A. 110 (42), 17035–17040. 10.1073/pnas.1302546110 24082121 PMC3801058

[B58] Ou Q. Jacobson Z. Abouleisa R. R. E. Tang X. L. Hindi S. M. Kumar A. (2019). Physiological biomimetic culture system for pig and human heart slices. Circ. Res. 125 (6), 628–642. 10.1161/CIRCRESAHA.119.314996 31310161 PMC6715512

[B59] Palombo P. Burkle A. Moreno-Villanueva M. (2022). Culture medium-dependent isoproterenol stability and its impact on DNA strand breaks formation and repair. Chem. Biol. Interact. 357, 109877. 10.1016/j.cbi.2022.109877 35276129

[B60] Pape C. Beier T. Li P. Jain V. Bock D. D. Kreshuk A. (2017). “Solving large multicut problems for connectomics via domain decomposition,” in 2017 IEEE International Conference on Computer Vision Workshops (ICCVW), Venice, Italy, Oct. 22 2017 to Oct. 29 2017, 1–10.

[B61] Parikh S. S. Blackwell D. J. Gomez-Hurtado N. Frisk M. Wang L. Kim K. (2017). Thyroid and glucocorticoid hormones promote functional T-tubule development in human-induced pluripotent stem cell-derived cardiomyocytes. Circ. Res. 121 (12), 1323–1330. 10.1161/CIRCRESAHA.117.311920 28974554 PMC5722667

[B62] Perbellini F. Thum T. (2020). Living myocardial slices: a novel multicellular model for cardiac translational research. Eur. Heart J. 41 (25), 2405–2408. 10.1093/eurheartj/ehz779 31711161 PMC7327529

[B63] Pfeuffer A.-K. M. Küpfer L. K. Shankar T. S. Drakos S. G. Volk T. Seidel T. (2023). Ryanodine receptor staining identifies viable cardiomyocytes in human and rabbit cardiac tissue slices. Int. J. Mol. Sci. 24 (17), 13514. 10.3390/ijms241713514 37686327 PMC10488113

[B64] Pitoulis F. G. Nunez-Toldra R. Xiao K. Kit-Anan W. Mitzka S. Jabbour R. J. (2022). Remodelling of adult cardiac tissue subjected to physiological and pathological mechanical load *in vitro* . Cardiovasc Res. 118 (3), 814–827. 10.1093/cvr/cvab084 33723566 PMC8859636

[B65] Poch C. M. Foo K. S. De Angelis M. T. Jennbacken K. Santamaria G. Bahr A. (2022). Migratory and anti-fibrotic programmes define the regenerative potential of human cardiac progenitors. Nat. Cell Biol. 24 (5), 659–671. 10.1038/s41556-022-00899-8 35550611 PMC9106586

[B66] Pogwizd S. M. Bers D. M. (2008). Rabbit models of heart disease. Drug Discov. Today Dis. Models 5 (3), 185–193. 10.1016/j.ddmod.2009.02.001 32288771 PMC7105925

[B67] Pucci E. Chiovato L. Pinchera A. (2000). Thyroid and lipid metabolism. Int. J. Obes. Relat. Metab. Disord. 24 (Suppl. 2), S109–S112. 10.1038/sj.ijo.0801292 10997623

[B68] Qiao Y. Dong Q. Li B. Obaid S. Miccile C. Yin R. T. (2019). Multiparametric slice culture platform for the investigation of human cardiac tissue physiology. Prog. Biophys. Mol. Biol. 144, 139–150. 10.1016/j.pbiomolbio.2018.06.001 29960680

[B69] Raekallio M. Ansah O. B. Kuusela E. Vainio O. (2002). Some factors influencing the level of clinical sedation induced by medetomidine in rabbits. J. Vet. Pharmacol. Ther. 25 (1), 39–42. 10.1046/j.1365-2885.2002.00382.x 11874525

[B70] Rich R. L. Day Y. S. Morton T. A. Myszka D. G. (2001). High-resolution and high-throughput protocols for measuring drug/human serum albumin interactions using BIACORE. Anal. Biochem. 296 (2), 197–207. 10.1006/abio.2001.5314 11554715

[B71] Rog-Zielinska E. A. Thomson A. Kenyon C. J. Brownstein D. G. Moran C. M. Szumska D. (2013). Glucocorticoid receptor is required for foetal heart maturation. Hum. Mol. Genet. 22 (16), 3269–3282. 10.1093/hmg/ddt182 23595884

[B72] Schram G. Pourrier M. Melnyk P. Nattel S. (2002). Differential distribution of cardiac ion channel expression as a basis for regional specialization in electrical function. Circulation Res. 90 (9), 939–950. 10.1161/01.RES.0000018627.89528.6F 12016259

[B73] Seabold S. Statsmodels P. (2010). “Econometric and statistical modeling with python,” in Proceedings of the 9th Python in science conference, Austin, Texas, June 28–30, 2010, 57–61.

[B74] Seidel T. Edelmann J. C. Sachse F. B. (2016). Analyzing remodeling of cardiac tissue: a comprehensive approach based on confocal microscopy and 3D reconstructions. Ann. Biomed. Eng. 44 (5), 1436–1448. 10.1007/s10439-015-1465-6 26399990 PMC4805509

[B75] Seidel T. Fiegle D. J. Baur T. J. Ritzer A. Nay S. Heim C. (2019). Glucocorticoids preserve the t-tubular system in ventricular cardiomyocytes by upregulation of autophagic flux. Basic Res. Cardiol. 114 (6), 47. 10.1007/s00395-019-0758-6 31673803 PMC9380897

[B76] Shi R. Reichardt M. Fiegle D. J. Kupfer L. K. Czajka T. Sun Z. (2023). Contractility measurements for cardiotoxicity screening with ventricular myocardial slices of pigs. Cardiovasc Res. 119 (14), 2469–2481. 10.1093/cvr/cvad141 37934066 PMC10651213

[B77] Shi T. Moulton V. R. Lapchak P. H. Deng G. M. Dalle Lucca J. J. Tsokos G. C. (2009). Ischemia-mediated aggregation of the actin cytoskeleton is one of the major initial events resulting in ischemia-reperfusion injury. Am. J. Physiol. Gastrointest. Liver Physiol. 296 (2), G339–G347. 10.1152/ajpgi.90607.2008 19095765

[B78] Stapleton M. T. Fuchsbauer C. M. Allshire A. P. (1998). BDM drives protein dephosphorylation and inhibits adenine nucleotide exchange in cardiomyocytes. Am. J. Physiology-Heart Circulatory Physiology 275 (4), H1260–H1266. 10.1152/ajpheart.1998.275.4.h1260 9746474

[B79] Tian Q. Pahlavan S. Oleinikow K. Jung J. Ruppenthal S. Scholz A. (2012). Functional and morphological preservation of adult ventricular myocytes in culture by sub-micromolar cytochalasin D supplement. J. Mol. Cell Cardiol. 52 (1), 113–124. 10.1016/j.yjmcc.2011.09.001 21930133

[B80] van der Walt S. Schönberger J. L. Nunez-Iglesias J. Boulogne F. Warner J. D. Yager N. (2014). scikit-image: image processing in Python. PeerJ 2, e453. 10.7717/peerj.453 25024921 PMC4081273

[B81] Volpp M. Holzgrabe U. (2019). Determination of plasma protein binding for sympathomimetic drugs by means of ultrafiltration. Eur. J. Pharm. Sci. 127, 175–184. 10.1016/j.ejps.2018.10.027 30391401

[B82] Wang K. Lee P. Mirams G. R. Sarathchandra P. Borg T. K. Gavaghan D. J. (2015). Cardiac tissue slices: preparation, handling, and successful optical mapping. Am. J. Physiol. Heart Circ. Physiol. 308 (9), H1112–H1125. 10.1152/ajpheart.00556.2014 25595366 PMC4551126

[B83] Watson S. A. Duff J. Bardi I. Zabielska M. Atanur S. S. Jabbour R. J. (2019). Biomimetic electromechanical stimulation to maintain adult myocardial slices *in vitro* . Nat. Commun. 10 (1), 2168. 10.1038/s41467-019-10175-3 31092830 PMC6520377

[B84] Yabu Y. Miyai K. Kobayashi A. Miki K. Doi K. Takamatsu J. (1987). A new type of albumin with predominantly increased binding affinity for 3,3’,5-triiodothyronine in a patient with Graves’ disease. J. Endocrinol. Invest. 10 (2), 163–169. 10.1007/BF03347183 3584855

