# Supplementary figures and images for: Effects of electro-mechanical uncouplers, hormonal stimulation and pacing rate on the stability and function of cultured rabbit myocardial slices
Source: Front Bioeng Biotechnol. 2024 Apr 5;12:1363538. doi: 10.3389/fbioe.2024.1363538 (PMC11026719; doi:10.3389/fbioe.2024.1363538)

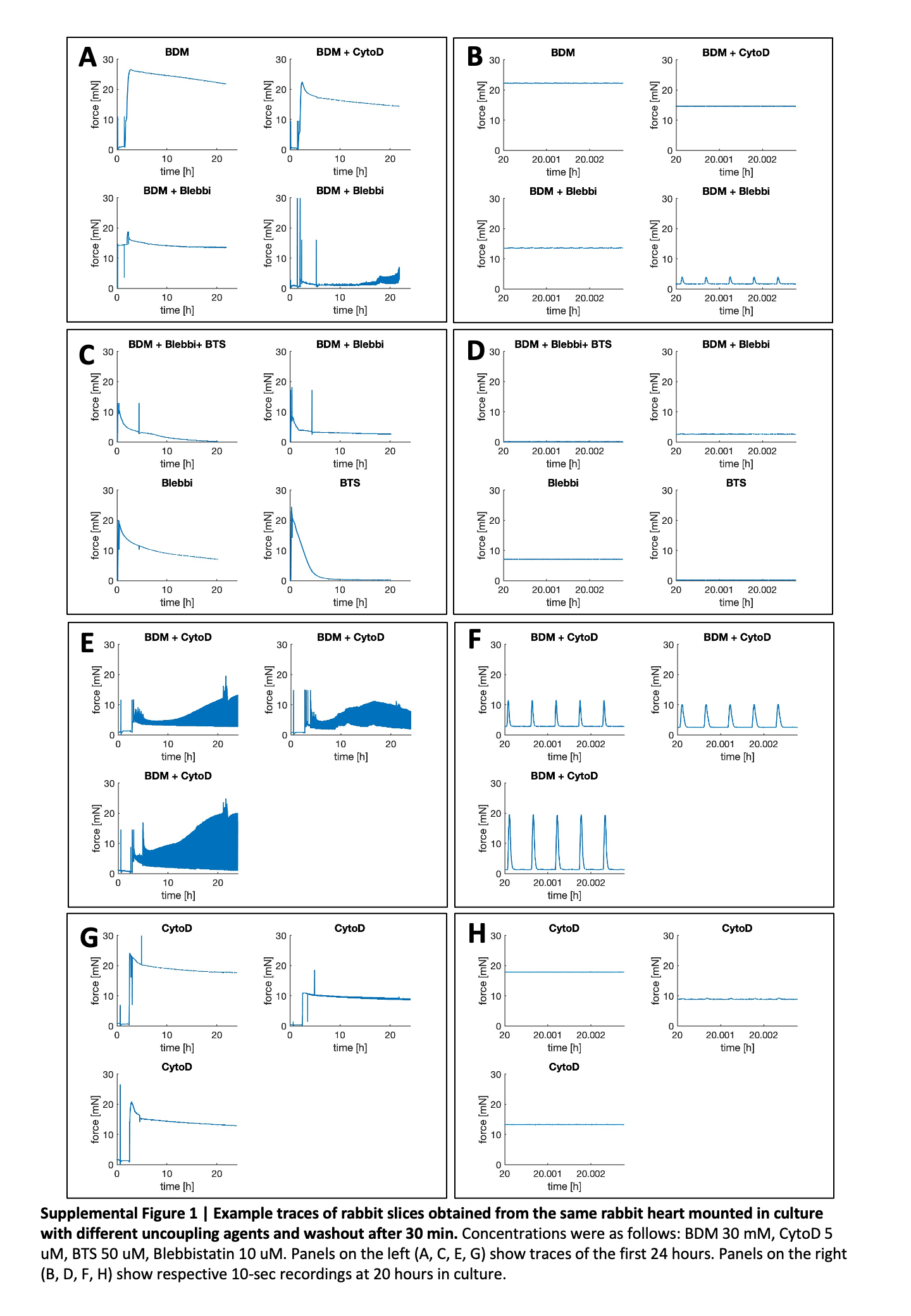

Supplement: Supplementary file 1 [file Image1.TIFF]

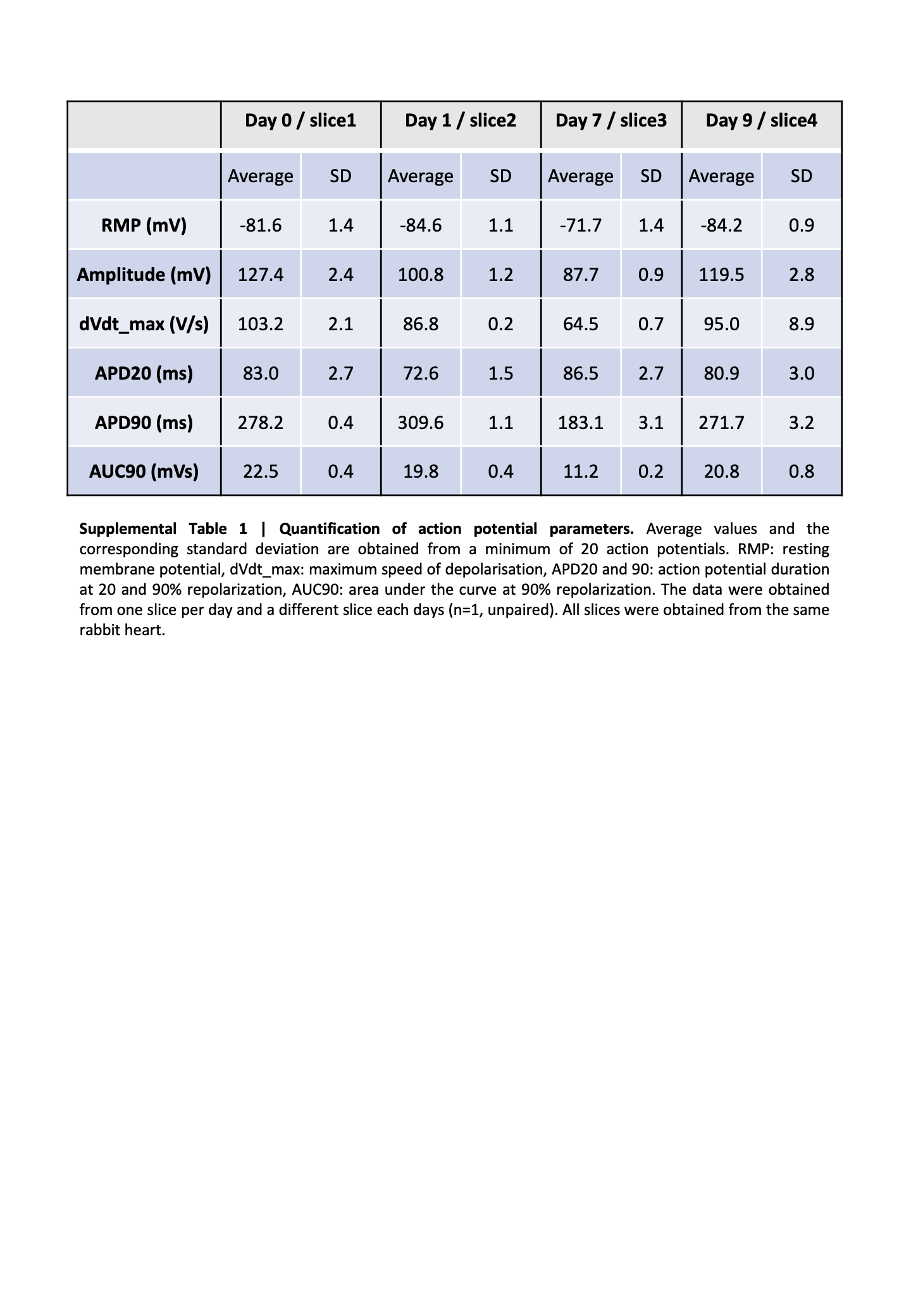

Supplement: Supplementary file 2 [file Image2.TIFF]
